# The Otago Exercise Program's effect on fall prevention: a systematic review and meta-analysis

**DOI:** 10.3389/fpubh.2025.1522952

**Published:** 2025-06-03

**Authors:** Chenyu Wang, Sung Min Kim

**Affiliations:** ^1^Department of Sport Science, Hanyang University, Seoul, Republic of Korea; ^2^Department of Physical Education, Human-Tech Convergence Program, Hanyang University, Seoul, Republic of Korea; ^3^Center for Artificial Intelligence Muscle, Hanyang University, Seoul, Republic of Korea

**Keywords:** Otago exercise, older adult, meta-analysis, fall, prevention

## Abstract

**Objectives:**

This study aims to compare the effectiveness of the Otago Exercise Program (OEP) in fall prevention between generally healthy older adults and those with compromised health conditions, assessing which group benefits more from the intervention.

**Design:**

This meta-analysis evaluated the effectiveness of the OEP in fall prevention among general older adults and older adults with compromised health, including individuals at high risk of falls, cognitive impairment, musculoskeletal disorders, or frailty syndrome.

**Methods:**

A comprehensive search was conducted in Web of Science, PubMed, Scopus, Cochrane Library, and Embase, following strict eligibility criteria. Data extraction, risk of bias assessment, and meta-analysis were conducted to evaluate the effectiveness of the intervention.

**Results:**

Fifteen studies with 1,278 participants were included. The OEP significantly improved balance (WMD = 0.15, 95% CI [–0.05, 0.35]), gait (WMD = 0.49, 95% CI [0.18, 0.80]), and lower limb strength (WMD = 0.84, 95% CI [0.61, 1.07]) in general older adults. The effects were more pronounced in older adults with compromised health, particularly in gait, particularly in gait (WMD = 0.92, 95% CI [0.13, 1.72]) and lower limb strength (WMD = 2.24, 95% CI [1.04, 3.45]). However, the OEP did not significantly improve physical function or upper limb strength in either group.

**Conclusion:**

The OEP effectively improves balance, gait, and lower limb strength, especially in older adults with compromised health. However, it does not significantly impact physical function or upper limb strength. This study has limitations, including potential bias, study heterogeneity, and variations in interventions, which may affect result reliability. A cautious interpretation is needed, and future research should focus on analyzing diverse populations and ensuring adequately sized samples to enhance the reliability of the findings.

**Systematic review registration:**

PROSPERO (CRD42024549302), https://www.crd.york.ac.uk/PROSPERO/view/CRD42024549302.

## 1 Introduction

As of 2023, the global population of individuals aged 60 years or older has reached approximately 1.1 billion, with projections indicating an increase to 1.4 billion by 2030 and 2.1 billion by 2050 (1). Falls are the second leading cause of accidental injury deaths globally (2), resulting in approximately 684,000 deaths annually, the majority of which occur among adults aged 60 years or older. Repeated falls can cause serious medical issues in older adults, Mgbeojedo et al. (3) including fractures and physical frailty, Kannus et al. (4) and Liston et al. (5) which may result in increased dependence on family members or assistive devices, fall-related anxiety, high economic costs, extended limitations on activity, and related issues (6, 7). Consequently, falls represent a significant risk to the wellbeing of older adults, highlighting the urgent need for effective interventions to mitigate this risk.

To mitigate the effect of falls among older adults, preventive interventions and education aimed at reducing fall risk are commonly implemented. Diverse exercise programs targeting flexibility, strength, balance, and endurance can improve mobility and physical function in older adults while reducing fall risk and related injuries (8, 9). OEP is a progressive training regimen specifically designed for older adults (10), emphasizing exercises that target balance and strength to reduce fall risk by improving physical stability (11). The OEP is a structured exercise program designed to enhance balance, strength, and mobility in older adults (12). It integrates lower limb exercises, such as single-leg stands and walking, with upper limb strength training, including arm curls, to promote overall functional stability and physical wellbeing (13). These exercises aim to strengthen core and lower limb muscles, improve coordination and reaction time, while enhancing stability during standing and walking (14, 15) By gradually increasing the difficulty and complexity of the exercises, the OEP effectively enhances the physical fitness and daily living abilities of older adults (16). The structured nature of this exercise program not only helps reduce the occurrence of falls but also significantly improves physical function in older adults, effectively lowering the risk of fall-related injuries (17).

However, the effectiveness of the OEP might vary between general older adults and those with compromised health, with existing research yielding inconsistent findings. In general older adults, studies have demonstrated significant benefits of OEP in improving mobility, physical function, and balance (18). Nonetheless, the OEP shows low to moderate certainty regarding its effectiveness in improving balance, mobility, and grip strength in frail or pre-frail older adults (19). The Otago Exercise Program enhances fall efficacy in older stroke survivors and has a positive impact on daily activities and quality of life. However, these effects do not reach statistical significance (20). When the OEP was implemented in individuals with dementia, four out of five participants improved from a higher to a lower fall risk category in at least one functional assessment measure (TUG, 30s-CST, 4-SBT, or BBS) (21). The OEP intervention for osteoarthritis (OA) led to significant improvements in balance, stability, and fall efficacy over six months. However, it did not effectively reduce the incidence of falls or prolong fall-free survival time (22). Other research suggests that the OEP has no significant effect on upper limb strength in older adults (23). These results suggest that the effectiveness of the OEP may vary based on health status, particularly in older adults with poorer health conditions, where the impact of the OEP may be limited. Therefore, this study aims to evaluate the effectiveness of the OEP in preventing falls among general older adults and those with compromised health, providing scientific evidence to optimize targeted intervention strategies.

Most existing research has primarily focused on the general older adult population, with limited comparative analysis of its effects across these two groups. It remains uncertain whether the OEP is more effective in preserving mobility and preventing falls among healthier older adults or in improving physical function and reducing fall risk in those with compromised health. Clarifying these distinctions is essential for refining fall prevention strategies tailored to diverse aging populations. Further research is needed to compare the effects of the OEP between general older adults and older adults with compromised health.

To address this, the present study systematically evaluates the effectiveness of the Otago Exercise Program (OEP) in improving fall-related outcomes and physical function through a meta-analysis, comparing its effects between two distinct groups: (1) generally healthy older adults and (2) older adults with compromised health conditions. Specifically, we examine whether OEP is more effective in preventing falls and enhancing physical function in generally healthy older adults or in reducing fall risk and improving physical function in older adults with compromised health conditions.

## 2 Methodology

This study is based on the PICOS framework and follows the Preferred Reporting Items for Systematic Reviews and Meta-Analyses (PRISMA 2020) standards and has been retrospectively registered on the PROSPERO platform (CRD42024549302).

Meta-analysis was conducted using Review Manager (RevMan 5.4) and Stata 18.0. Effect sizes were reported as weighted mean differences (WMD) with 95% confidence intervals (CI), as all included studies measured the same continuous outcomes using a consistent unit of measurement. This approach enables direct comparison of effect sizes without standardization, preserving clinical interpretability and providing a clearer understanding of the intervention's impact. A random-effects model was employed to account for potential heterogeneity, which was assessed using I^2^ statistic, with *I*^2^>50% indicating moderate-to-high heterogeneity.

Potential publication bias was assessed through funnel plots and Egger's regression test (24). To further adjust for potential publication bias, the Trim-and-Fill method was applied (25). This non-parametric statistical method estimates the number of missing studies due to publication bias and imputes them to produce an adjusted overall effect size. The method helps determine whether the observed results are robust even after accounting for potentially missing studies. Data visualization incorporated forest plots to illustrate effect estimates with corresponding 95% CIs for each study, while sensitivity analyses were conducted to evaluate the robustness of the findings.

We conducted subgroup analyses of the Otago Exercise Program (OEP) intervention to examine the effects of session duration and total intervention duration on its effectiveness. This analysis aimed to assess whether variations in intervention length influenced fall prevention outcomes. Due to the limited number of available studies, subgroup analyses based on health conditions (e.g., cognitive impairment, musculoskeletal disorders, and frailty syndrome) were not performed. Future research should consider stratified analyses to further investigate potential differences in OEP effectiveness across these specific health conditions.

### 2.1 Literature inclusion and exclusion criteria

#### 2.1.1 Inclusion criteria

(1) This study included older adults aged ≥ 60 years, categorized into two groups: General older adults: Individuals without chronic diseases, mobility impairments, and with no cognitive impairment (MMSE ≥ 24). (2) Older adults with compromised health: Individuals with at least one of the following conditions-history of falls, cognitive impairment (defined as MMSE < 24, or where MMSE was not specified in the original study), musculoskeletal disorders, or frailty syndrome. Studies were included only if they reported fall-related outcome measures and met predefined selection criteria.

#### 2.1.2 Exclusion criteria

(1) Duplicate publications or redundant studies reporting the same data. (2) Review articles, meta-analyses, or conference abstracts that did not contain original data. (3) Studies lacking extractable data or failing to report relevant fall-related outcomes. (4) Studies with poor methodological quality, as determined by a risk of bias assessment. (5) Studies on the OEP that did not assess fall prevention or fall-related parameters. (6) Studies without a control group, including single-arm intervention studies and observational studies without a comparator. (7) Studies involving hospitalized older adults or those in long-term care facilities, as their rehabilitation settings differ from those of community-dwelling older adults. (8) Studies including older adults with severe health conditions that could significantly limit their ability to participate in exercise interventions, such as advanced neurodegenerative diseases (e.g., late-stage Alzheimer's or Parkinson's disease), terminal illnesses, or severe cardiovascular conditions. The search strategy, inclusion criteria, screening process, and exclusion criteria were established in accordance with PRISMA guidelines.

### 2.2 Literature search strategy

Searches were conducted in the Web of Science, PubMed, Scopus, Cochrane Library, and Embase databases using the Boolean search terms (“old people” OR “older adult” OR “senior” OR “senior citizen” OR “aged” OR “senior adult” OR “old-age person” OR “older adult”) AND (“Otago exercise” OR “Otago”). The search was conducted up to July 1, 2024. Moreover, the reference lists of included studies were hand-searched to detect any possibly missed studies, and the references of pertinent studies were also traced.

### 2.3 Literature screening and data extraction

Two researchers performed the literature screening and data extraction. Data extracted from all selected RCTs included: detailed participant information (characteristics, sample size, age, and gender), intervention characteristics (type and frequency), and outcome measures. Balance was measured using the Berg Balance Scale (BBS) (26); physical function was evaluated with the Short Physical Performance Battery (SPPB) (27); gait was evaluated by the 6-Min Walk Test (6MWT) (28); lower limb strength was assessed using the 30-Second Chair Stand Test (30s CST) (29); mobility was evaluated with the Timed Up and Go Test (TUG) (30); and upper limb strength was measured by grip strength tests (HGR, HGL) (31).

### 2.4 Bias risk assessment of included studies

Two researchers used the Cochrane Handbook for Systematic Reviews of Interventions to evaluate the standard of the selected studies. For each study, judgments were made on the following seven factors: random sequence generation, allocation concealment, blinding of participants and personnel, blinding of outcome assessment, missing outcome data, selective reporting, and other bias sources. Each aspect was rated as “Yes” (low risk), “No” (high risk), or “Unclear.” The greater the number of “low risk” ratings, the lower the potential for publication bias and the higher the study quality.

### 2.5 Statistical analysis

Data were analyzed using Stata 17.0 software, and a meta-analysis was conducted. Heterogeneity of the included studies was tested; if *I*^2^ < 50%, the studies were deemed homogeneous, and a fixed-effects model was applied for meta-analysis. If *I*^2^≥50%, the studies were considered heterogeneous, and a random-effects model was applied for meta-analysis, along with an analysis of the sources of heterogeneity. Sensitivity analysis and Egger's regression intercept test were used to evaluate the robustness of the results and assess publication bias.

## 3 Research results

### 3.1 Basic characteristics of included studies

**Figure 1:**
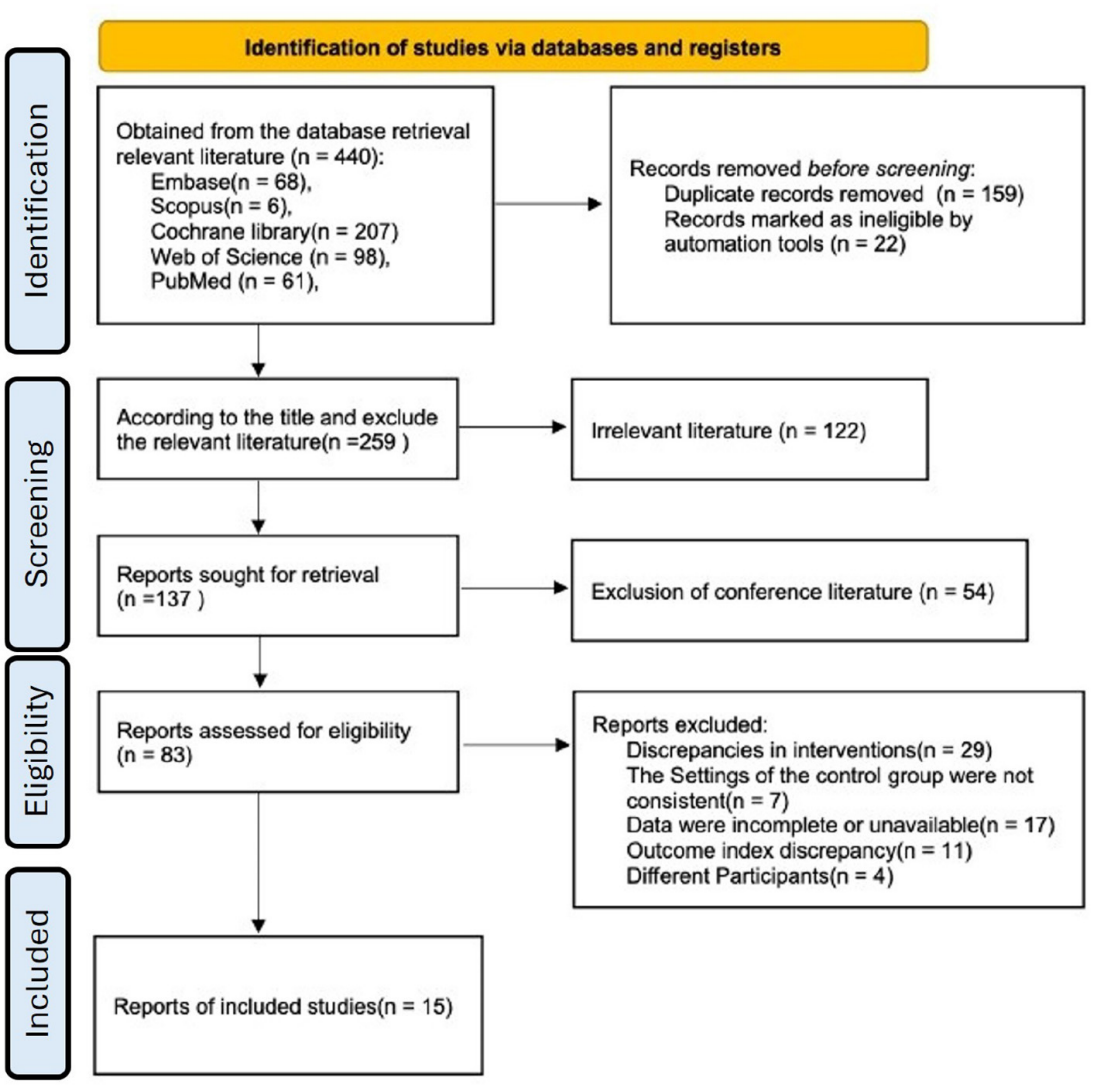
Screening flowchart.

A total of 440 articles were collected from database searches and other sources, including 159 duplicates and 22 records marked as non-eligible (review articles) by the automation tool (EndNote20). This left 259 studies for further examination. After reviewing titles and abstracts, 176 studies were excluded for not meeting the criteria, leaving 83 studies for full-text review. After reviewing the full text, 15 studies were included in the meta-analysis (Figure 1).

A total of 15 RCTs included 696 participants in the intervention group and 582 participants in the control group, with a combined sample size of 1,278 participants, ranging from 15 to 160 per study. The participants were older adults aged between 60 and 92 years. Eight studies applied OEP to the general older adult population (14, 32–38), while seven studies applied OEP to older adults with high fall risk, previous fall experiences, cognitive impairment, or certain diseases (11, 39–44). Control group participants engaged in other exercise programs, such as walking (42), gaze stability exercises (GSE) combined with core muscle strengthening (6), or Tai Chi (37), Some control groups received usual care (11, 32–36, 39–41, 43, 44), while others maintained regular activities without any intervention (38). In the intervention group, three studies used a combination of OEP with other interventions: OEP plus core muscle strengthening (6), OEP plus oral nutritional supplements (41), and OEP plus motivational interviewing (32). All other studies used OEP alone, details can be seen in Table 1.

**Table 1:** Basic information of literature.

**Study**	**Participants**	**Intervention characterization**	**Outcomes of interest/results**
García-Gollarte et al. (41)	Frail older adults, EG/CG: 39/34	EG: Otago exercise, CG: Regular care	BBS, TUG, SPPB, 6MWT
Jahanpeyma et al. (42)	Older adults at high risk of falling, EG/CG: 35/36	EG: Otago exercise, CG: Walking training	BBS, 6MWT, 30CST
Liew et al. (43)	Older adults with a history of falls in the past year, EG/CG: 34/33	EG: Otago exercise, CG: Regular care	TUG, HGS-R, HGS-L
Liu-Ambrose et al. (11)	Older adults at high risk of falling, EG/CG: 31/28	EG: Otago exercise, CG: Regular care	TUG
Xiao et al. (44)	Older Adults Undergoing Hip Fracture Replacement (HFR) Patients, EG/CG: 38/39	EG: Otago exercise, CG: Regular care	TUG
Chen et al. (40)	Older adults with cognitive impairment, EG/CG: 31/31	EG: Otago exercise, CG: Regular care	BBS, TUG
Bjerk et al. (39)	Older adults with a history of falls in the past year, EG/CG: 77/78	EG: Otago exercise, CG: Regular care	BBS, 30CST
Johnson et al. (34)	Older, EG/CG: 61/56	EG: Otago exercise + Motivational interviewing, CG: Regular care	SPPB
Lytras et al. (36)	Older, EG/CG: 75/75	EG: Otago exercise, CG: Regular care	BBS, TUG, 30CST
Arkkukangas et al. (32)	Older, EG/CG: 61/56	EG: Otago exercise, CG: Regular care	SPPB, HGS-R, HGS-L
Benavent-Caballer et al. (38)	Older, EG/CG: 28/23	EG: Otago exercise, CG: No intervention	BBS, TUG, SPPB, 6MWT
Son et al. (37)	Older, EG/CG: 24/26	EG: Otago exercise, CG: Tai Chi	TUG, 30CST
Kp et al. (6)	Older, EG/CG: 15/15	EG1: Otago exercise, EG2: Gaze stability exercise	BBS
Genç and Bilgili (33)	Older, EG/CG: 28/28	EG: Otago exercise, CG: Regular care	BBS, HGS-R, HGS-L, 6MWT, 30CST
Kocic et al. (35)	Older, EG/CG: 38/39	EG: Otago exercise, CG: Regular care	TUG

### 3.2 Quality assessment of included studies

The assessment was performed using the Cochrane Risk of Bias Tool, evaluating seven domains: random sequence generation (selection bias), allocation concealment (selection bias), blinding of participants and personnel (performance bias), blinding of outcome assessment (detection bias), incomplete outcome data (attrition bias), selective reporting (reporting bias), and other biases (45).

**Figure 2:**
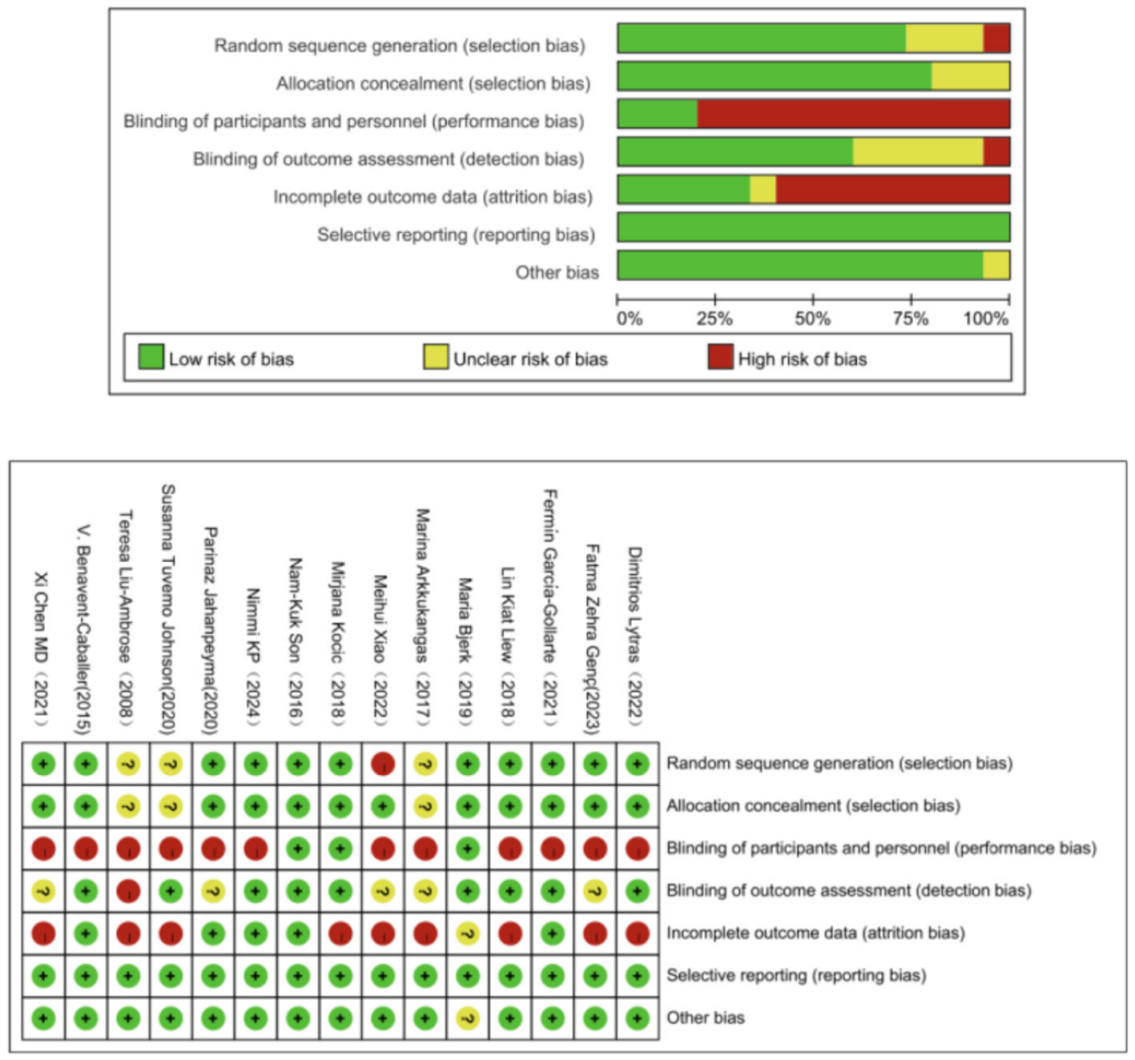
Risk of bias assessment.

The evaluation of quality of the included studies revealed that, although most studies employed sound methods for randomization and concealment of allocation, three studies stated random allocation but failed to provide detailed information on the randomization process (11, 32, 34), and were therefore rated as having a moderate bias risk. One study used hospital admission order as the randomization method (44), and was therefore rated as having a high bias risk. Within the included studies, one was not an RCT (35). There were deficiencies in the implementation of blinding and handling of missing data. Several studies did not implement blinding (11, 14, 32–34, 36–38, 40–43), and were therefore assessed as having a high risk of bias. Two studies implemented single-blinding (39) and double-blinding (35), and were therefore rated as having a low bias risk (Figure 2).

Studies were classified as high, moderate, or low quality using the Cochrane Risk of Bias Tool. Low-quality studies were defined as those exhibiting a high risk of bias in three or more key domains, severe methodological flaws (e.g., non-randomized design, inadequate control groups), or small sample sizes that compromised statistical power. Such studies were excluded to ensure the reliability and validity of the results.

The quality assessment of the included studies confirmed that none met the exclusion criteria for low quality. While some exhibited moderate risk of bias (e.g., unclear randomization, lack of blinding), all adhered to essential methodological standards and were deemed appropriate for inclusion in the meta-analysis.

### 3.3 Effect of OEP on balance in older adults

**Figure 3:**
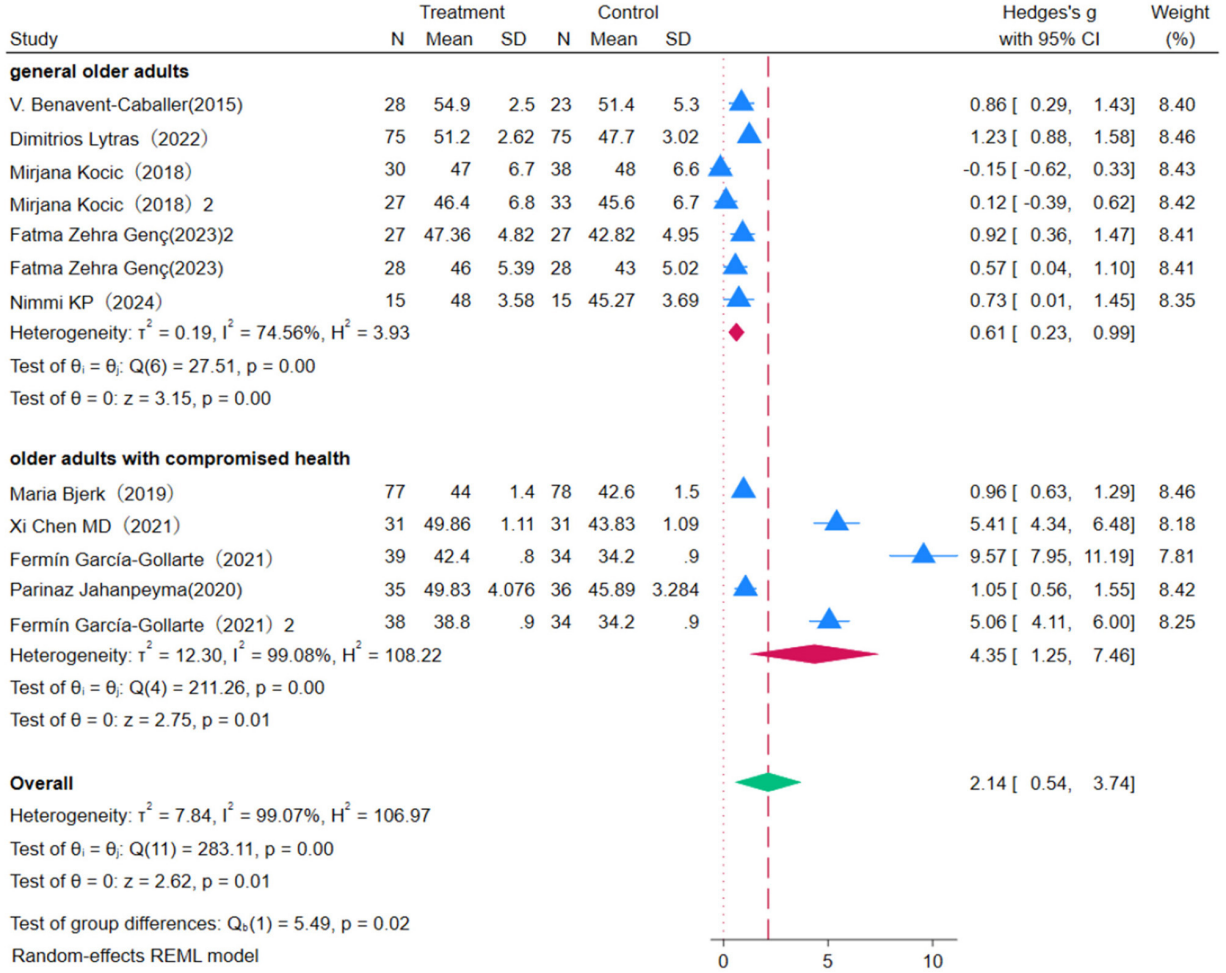
The impact of the OEP on improving balance in older adults.

Eight studies were included to evaluate the impact of OEP intervention on balance in older adults, using the Berg Balance Scale (BBS) as the assessment metric (Figure 3). Due to high heterogeneity among studies (*P* = 0.00, *I*^2^ = 99.07%), a random-effects model was used for the meta-analysis. The results showed that the OEP program significantly improves balance in older adults [WMD = 2.14, 95% CI (0.54, 3.74), *p* = 0.00], though the effects varied among different populations. Specifically, the effect was more pronounced in older adults with compromised health [WMD = 0.61, 95% CI (0.23, 0.99)] and in the general older adult population [WMD = 4.35, 95% CI (1.25, 7.46)]. This indicates that populations with poorer baseline balance have greater potential for improvement. The *p*-value of 0.02 and *p* < 0.05 indicate a statistically significant difference between the two groups, suggesting that older adults with compromised health showed more significant improvements in balance following OEP intervention relative to the general older adult population. Given the high heterogeneity in the meta-analysis, sensitivity analysis showed that excluding the majority of studies did not notably change the effect size estimates or confidence intervals, indicating the robustness of the meta-analysis results. Egger's regression intercept test showed notable reporting bias (*p* = 0.002). To further assess the reliability of the findings, the Trim-and-Fill method was applied to identify and adjust for potential publication bias. After imputing the missing studies, the combined effect size was slightly reduced but remained statistically significant, indicating the robustness of the results.

**Figure 4:**
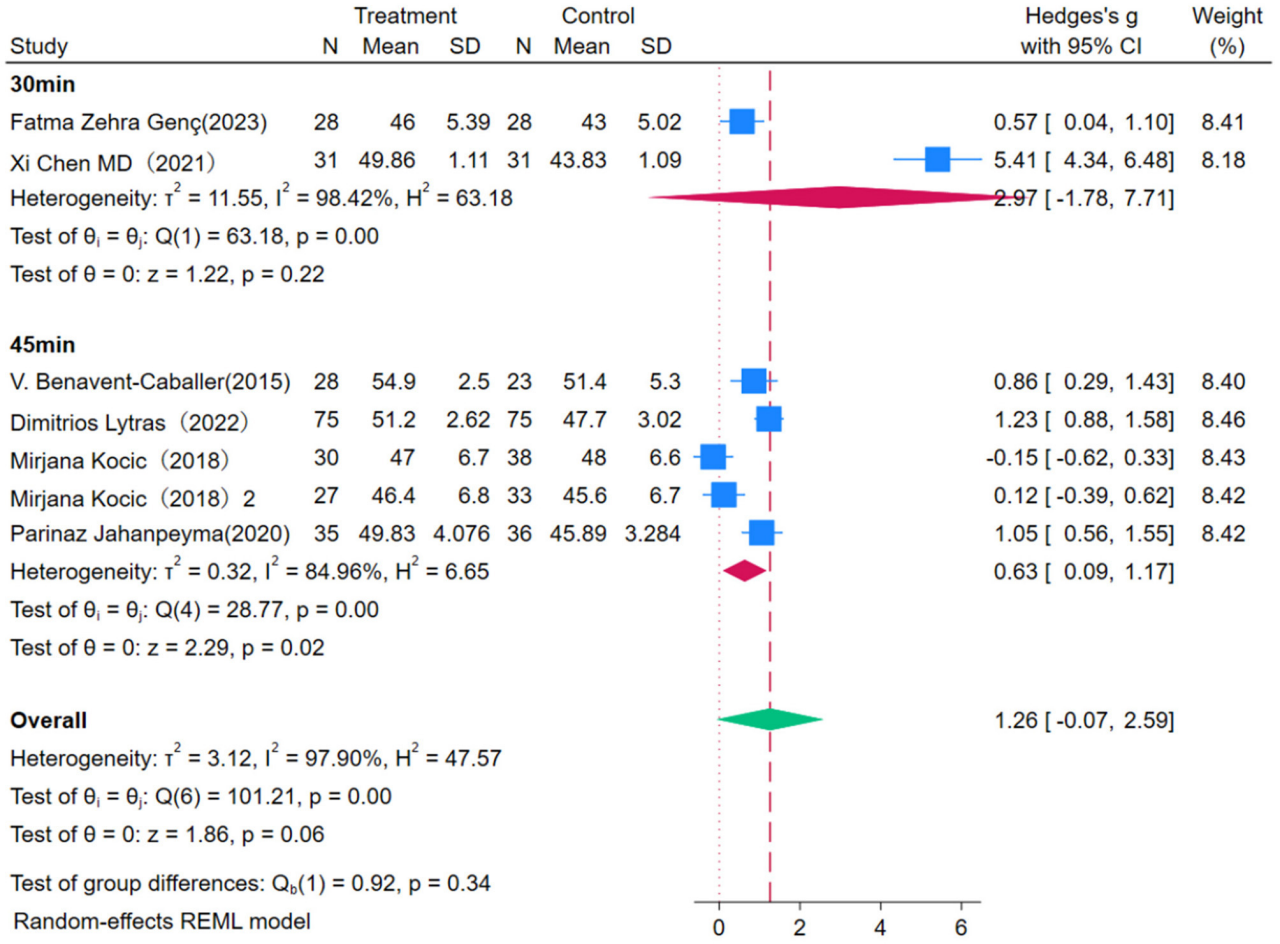
Subgroup analysis results for balance.

**Figure 5:**
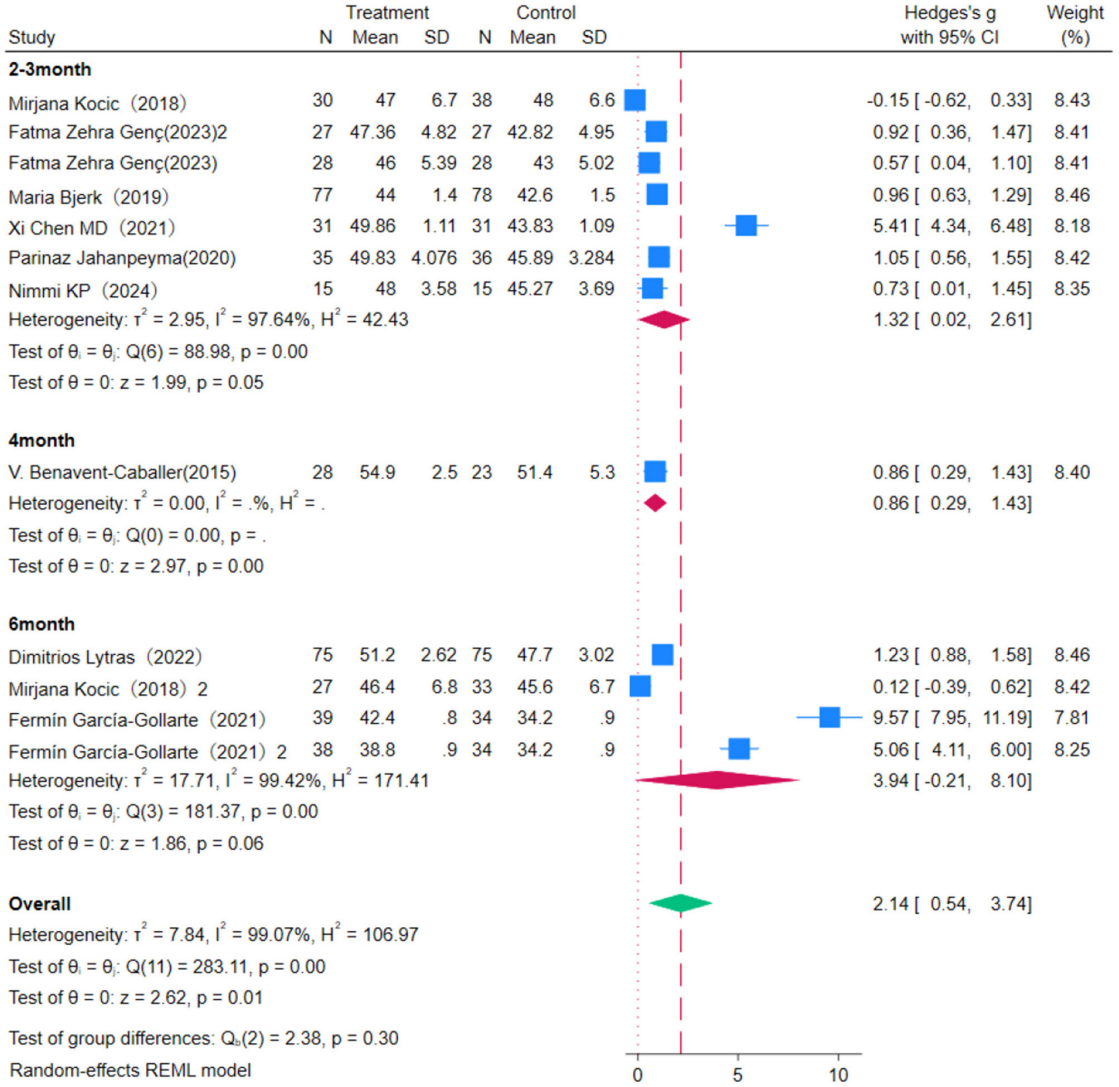
Subgroup analysis results for balance.

This study conducted a subgroup analysis on the effect of OEP intervention duration on balance improvement in older adults (Figures 4, 5). The overall effect size [WMD = 2.14, 95% CI (0.54, 3.74)], indicating a positive impact of OEP on balance ability. Among different intervention durations, the 4 month intervention showed the most stable effect [WMD = 0.86, 95% CI (0.29, 1.43)], whereas the 2–3 month [WMD = 1.32, 95% CI (0.02, 2.61)] and 6 month [WMD = 3.94, 95% CI (–0.21, 8.10)] groups exhibited high heterogeneity, making the results less robust. Interventions with 45 minutes of exercise per session were more effective in improving balance in older adults [WMD = 0.63, 95% CI (0.09, 1.17)], whereas 30-min sessions, although statistically significant [WMD = 2.97, 95% CI (–1.78, 7.71)], had a wider confidence interval including zero, making the effect statistically non-significant. Therefore, it is recommended to consider extending exercise duration in clinical practice to enhance effectiveness.

### 3.4 Impact of Otago Exercise Program on older adults' physical function

**Figure 6:**
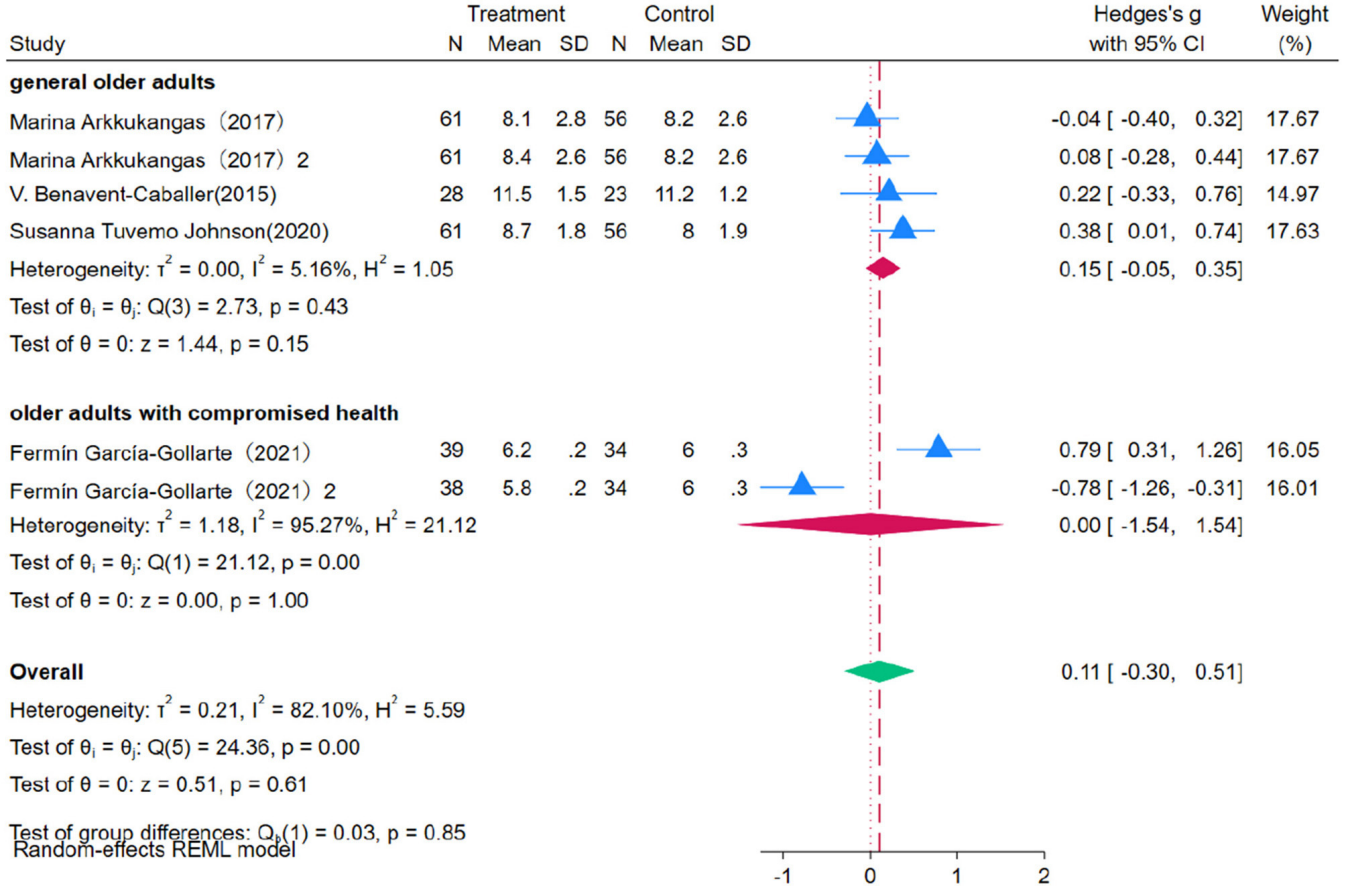
The impact of the OEP on improving physical function in older adults.

Four studies evaluating the impact of OEP intervention on older adults' physical function used SPPB as the assessment measure. Due to substantial heterogeneity among studies (*P* = 0.00, *I*^2^ = 82.10%), a random-effects model was used for the meta-analysis (Figure 6). The results indicate that OEP has a marginal impact on the overall physical function of older adults [WMD = 0.11, 95% CI (−0.05, 0.35)], with a wide confidence interval that includes zero, making the effect statistically non-significant. The effect on older adults with compromised health [WMD = 0.00, 95% CI (−1.54, 1.54)] and on the general older adult population [WMD = 0.15, 95% CI (−0.05, 0.35)] was also non-significant. This lack of significance may be due to insufficient sample sizes. Future research should increase sample size and diversity to further validate and expand these findings. Given the high heterogeneity, sensitivity analysis was conducted to assess the impact of each study on the overall results. The results indicated that the exclusion of most studies did not significantly alter the effect size estimates or confidence intervals, demonstrating the robustness of the meta-analysis findings. The meta-analysis results were not significantly affected by individual study biases. The Egger's regression intercept test indicated no significant publication bias (*p* = 0.957). However, the Trim-and-Fill method identified and imputed two potentially missing studies, indicating a certain degree of publication bias. However, after adjusting for these missing studies, the effect size remained statistically non-significant. This suggests that while some publication bias may be present, it is unlikely to have a substantial impact on the overall conclusion.

Subgroup analysis indicated that OEP did not significantly affect the Physical Function of older adults across different intervention durations and session lengths. To better understand the true impact of varying intervention durations, further high-quality, standardized studies are recommended.

### 3.5 Impact of OEP on older adults' gait

**Figure 7:**
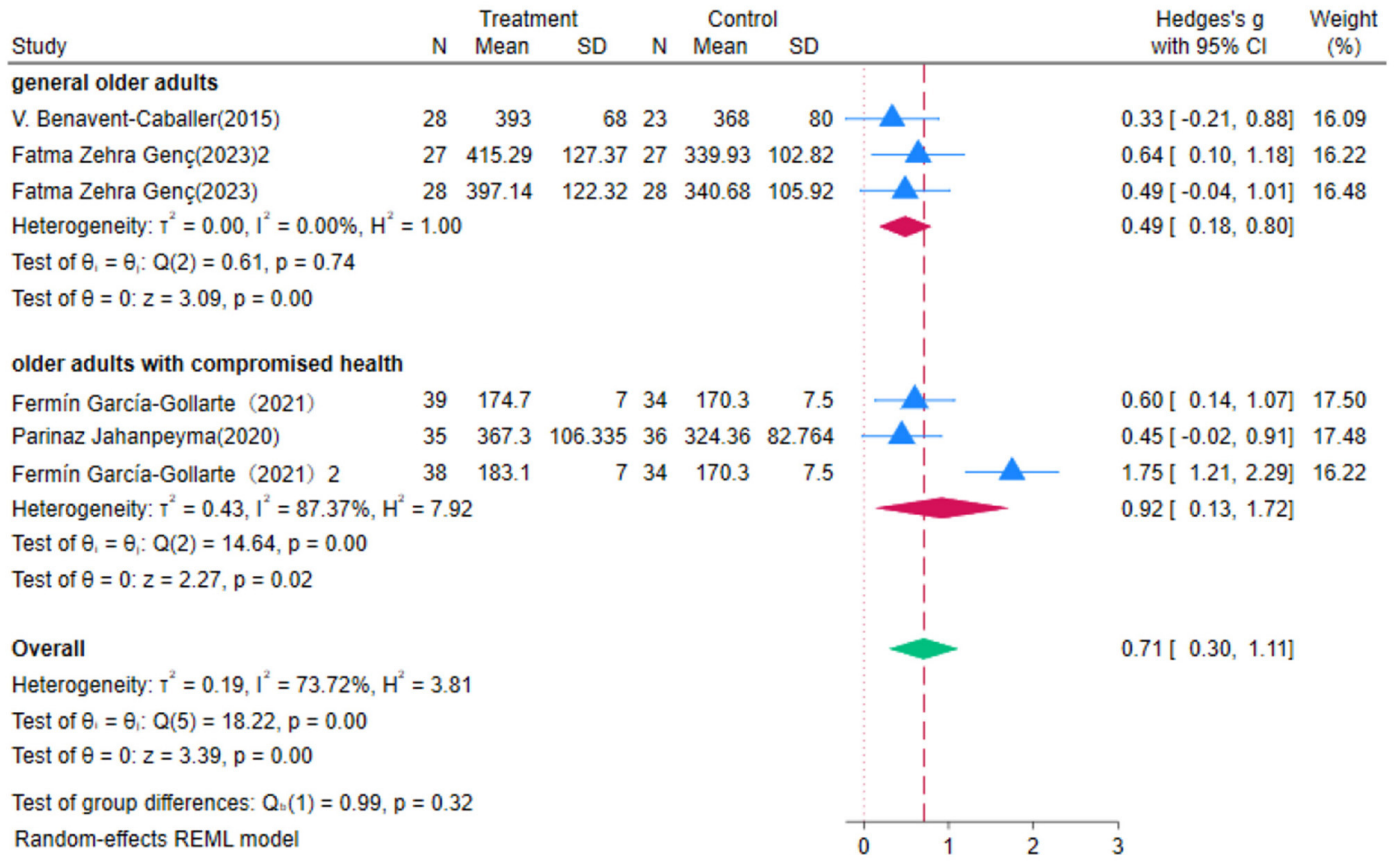
The impact of the OEP on improving gait in older adults.

Four studies evaluated the impact of OEP intervention on older adults' gait, using the 6MWT as the assessment measure. Due to substantial heterogeneity among studies (*P* = 0.00, *I*^2^ = 73.72%), a random-effects model was employed for the meta-analysis (Figure 7). The results showed that OEP significantly improved older adults' gait [WMD = 0.71, 95% CI (0.30, 1.11)]. The improvement in gait for general older adults [WMD = 0.49, 95% CI (0.18, 0.80)] was less pronounced compared to the improvement for older adults with compromised health [WMD = 0.92, 95% CI (0.13, 1.72)]. Given the high heterogeneity observed in the meta-analysis, sensitivity analysis revealed that excluding the majority of studies did not substantially alter the effect size estimates or confidence intervals, confirming the robustness of the findings. The Egger's regression intercept test indicated that publication bias was not significant (*p* = 0.573), suggesting that the results are reliable and representative. The Trim-and-Fill method identified and imputed two potentially missing studies, suggesting the presence of some publication bias. The adjusted effect size remained statistically significant, indicating that while some publication bias may exist, it does not substantially affect the overall conclusions, and the findings remain robust.

**Figure 8:**
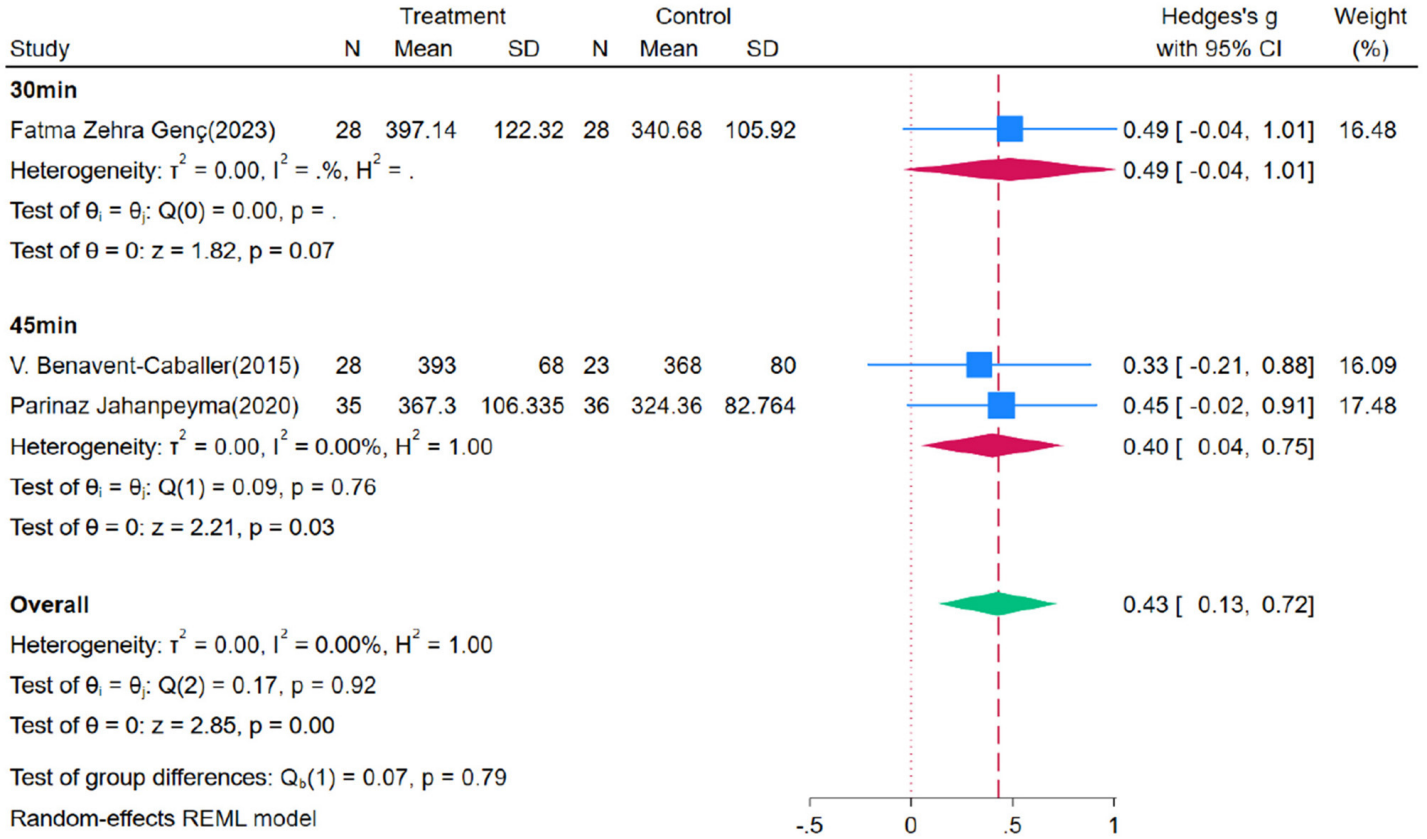
Subgroup analysis results for gait.

**Figure 9:**
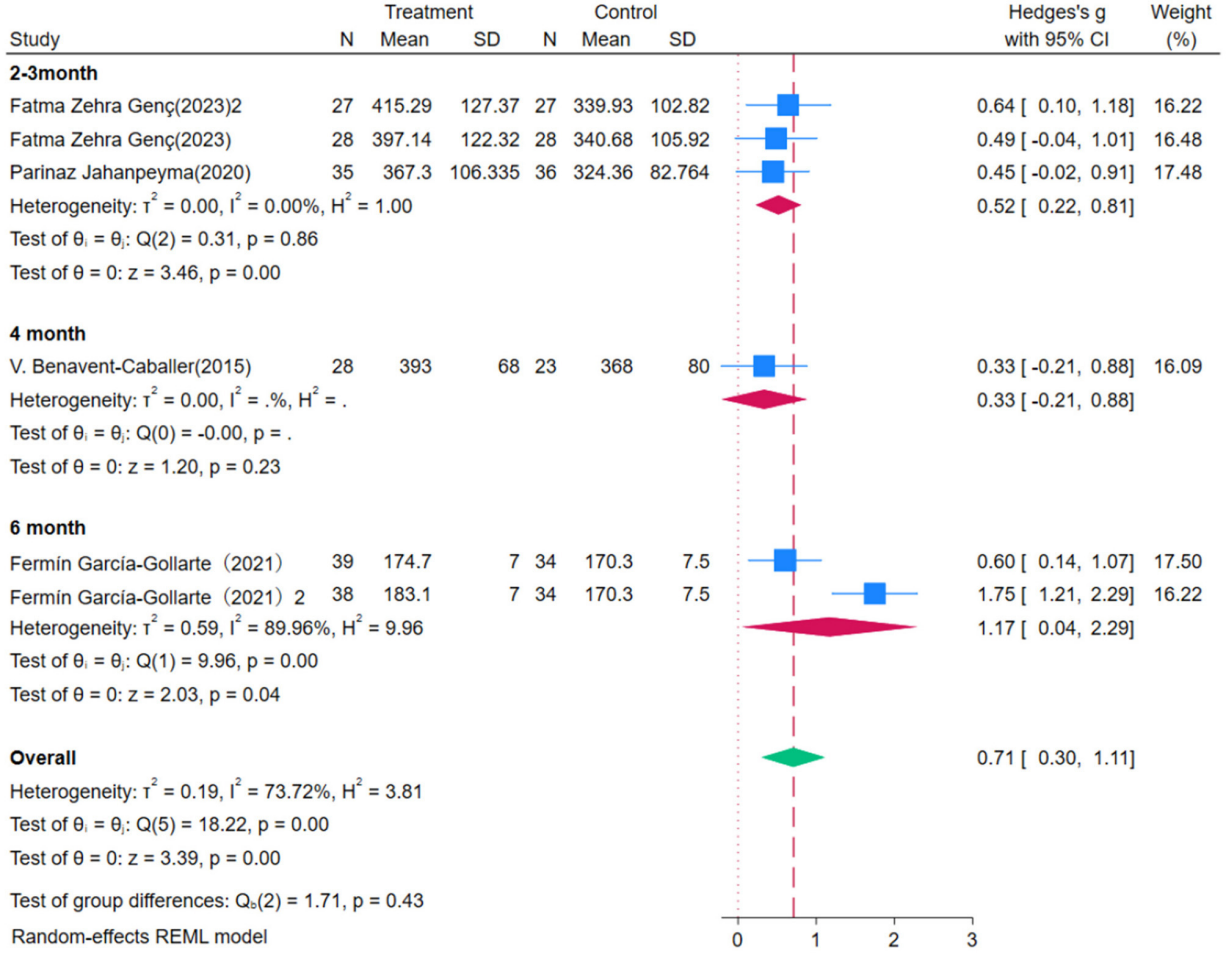
Subgroup analysis results for gait.

Through subgroup analysis, it was found that OEP is more effective for older adults who engage in long-term participation (Figures 8, 9). The effect of a six-month intervention [WMD = 0.89, 95% CI (0.05, 1.74), *p* = 0.00] is superior to that of a 2–3-month intervention [WMD = 0.52, 95% CI (0.22, 0.81), *p* = 0.86]. These findings suggest that a longer duration of OEP intervention may be more effective in improving gait among older adults, particularly those with compromised health. Clinical practice should consider extending the intervention duration to maximize its benefits and enhance overall effectiveness. For exercise duration per session, 30 min [WMD = 0.49, 95% CI (−0.04, 1.01)] has a broad confidence interval including zero, indicating statistical insignificance; while 45 minutes of exercise per session [WMD = 0.40, 95% CI (0.04, 0.75)] demonstrates a significant positive impact, suggesting that longer exercise duration may be more effective in improving gait in older adults. Although OEP significantly improves gait in older adults, the limited number of included studies necessitates cautious interpretation of the generalizability of these findings. Future research should aim for larger sample sizes and longer intervention durations to provide more robust evidence.

### 3.6 The impact of the OEP on improving lower limb strength in older adults

**Figure 10:**
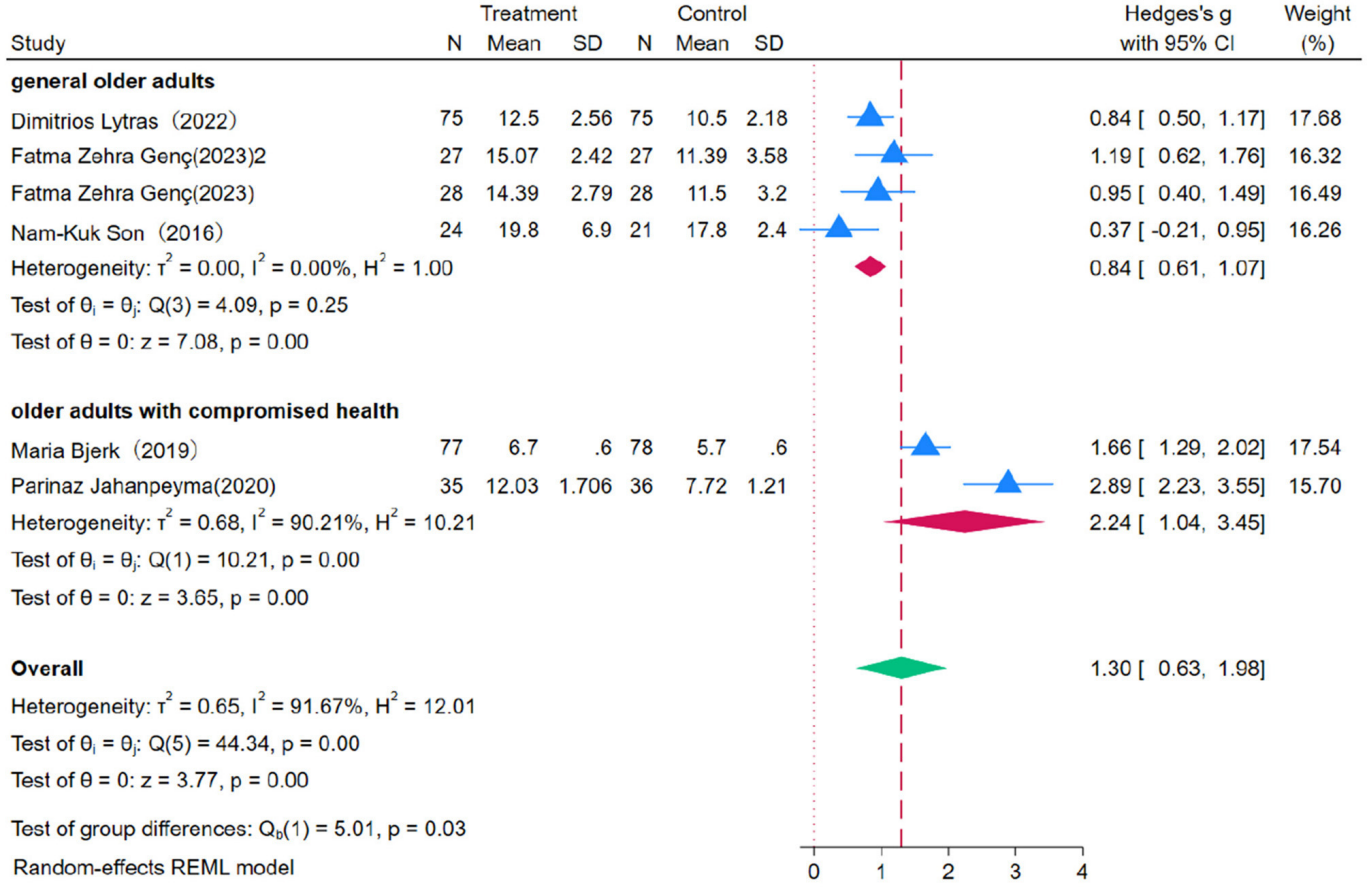
The impact of the OEP on improving lower limb strength in older adults.

Four studies were included to evaluate the effect of OEP intervention on lower limb strength in the older adult, using the 30s CST as the assessment metric. Due to substantial heterogeneity among the studies (*P* = 0.00, *I*^2^ = 91.67%), a random-effects model was employed for the meta-analysis (Figure 10). The results indicate that OEP significantly improves lower limb strength in older adults [WMD = 1.30, 95% CI (0.63, 1.98)]. OEP intervention demonstrates a more pronounced effect on improving lower limb strength in older adults with compromised health [WMD = 2.24, 95% CI (1.04, 3.45)] compared to the general older adult population [WMD = 0.84, 95% CI (0.61, 1.07)]. The statistical significance (*P* = 0.03, *p* < 0.05) indicates that the difference in effect size between the two groups is significant, suggesting that older adults with compromised health experience greater improvements in lower limb strength following OEP intervention compared to the general older adult population. Funnel plot analysis indicated potential publication bias in the 30s CST, as evidenced by asymmetry and the presence of imputed studies identified through the Trim-and-Fill method. However, after adjusting for these missing studies, the overall effect size remained statistically significant, suggesting that while some degree of publication bias may exist, it does not substantially affect the robustness of the findings. Egger's regression intercept test indicated that publication bias was not significant (*p* = 0.545), suggesting that the results are reliable and representative.

**Figure 11:**
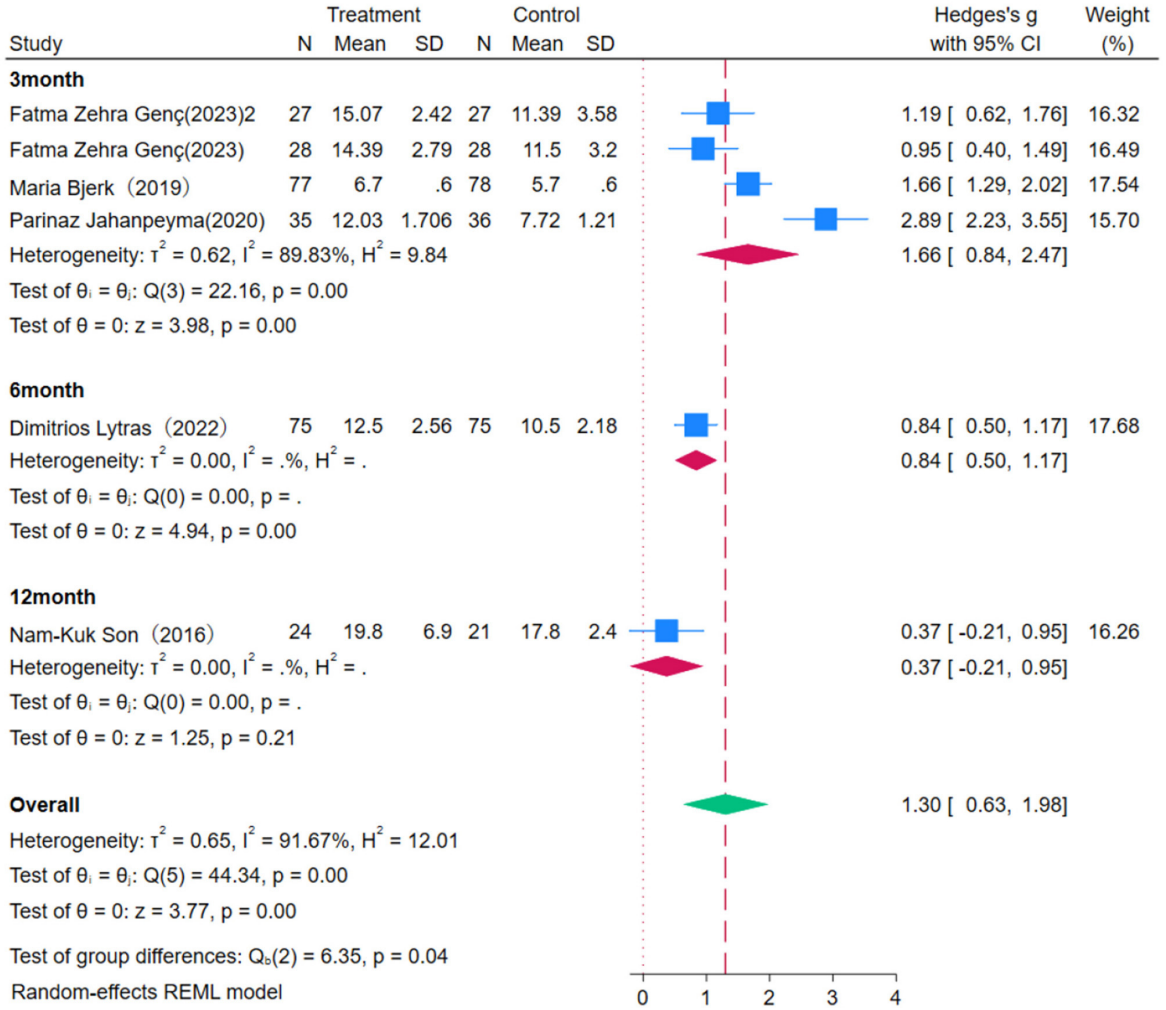
Subgroup analysis results for lower limb strength.

**Figure 12:**
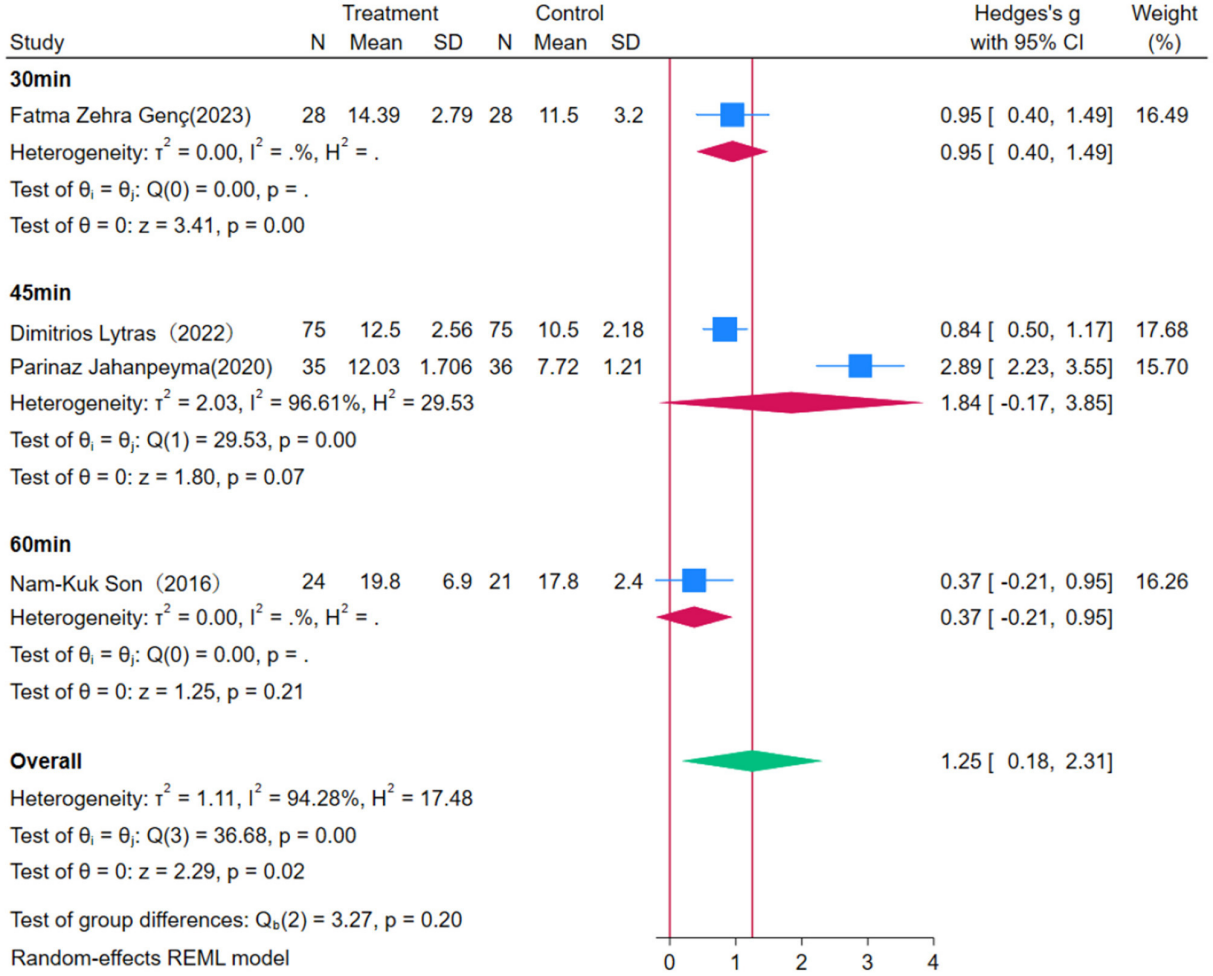
Subgroup analysis results for lower limb strength.

Subgroup analysis reveals that OEP significantly improves lower limb strength in older adults (Figures 11, 12), with a more pronounced effect observed in interventions lasting 2–3 months [WMD = 1.66, 95% CI (0.84, 2.47)] compared to those lasting six months [WMD = 0.84, 95% CI (0.50, 1.17)]. These differences suggest that short-term interventions may yield quicker results, while longer interventions might help in consolidating these gains. Regarding the duration of each exercise session, 30-min sessions [WMD = 0.95, 95% CI (0.40, 1.49)] were effective in improving lower limb strength. In contrast, 45-minute [WMD = 1.84, 95% CI (−1.84, 3.85)] and 60-min sessions [WMD = 0.37, 95% CI (−0.21, 0.95)] had wide confidence intervals that included zero, indicating statistically non-significant effects. The significant improvement with 30-min sessions could be attributed to the duration being short enough to avoid fatigue, thereby maximizing the exercise's benefits. This indicates that OEP is particularly effective under specific conditions, such as 30-min sessions, but the optimal exercise duration may vary depending on participant characteristics and specific study conditions. To achieve the best outcomes, it is recommended that exercise duration be adjusted in clinical practice according to individual needs and capabilities.

### 3.7 The impact of the OEP on improving mobility in older adults

**Figure 13:**
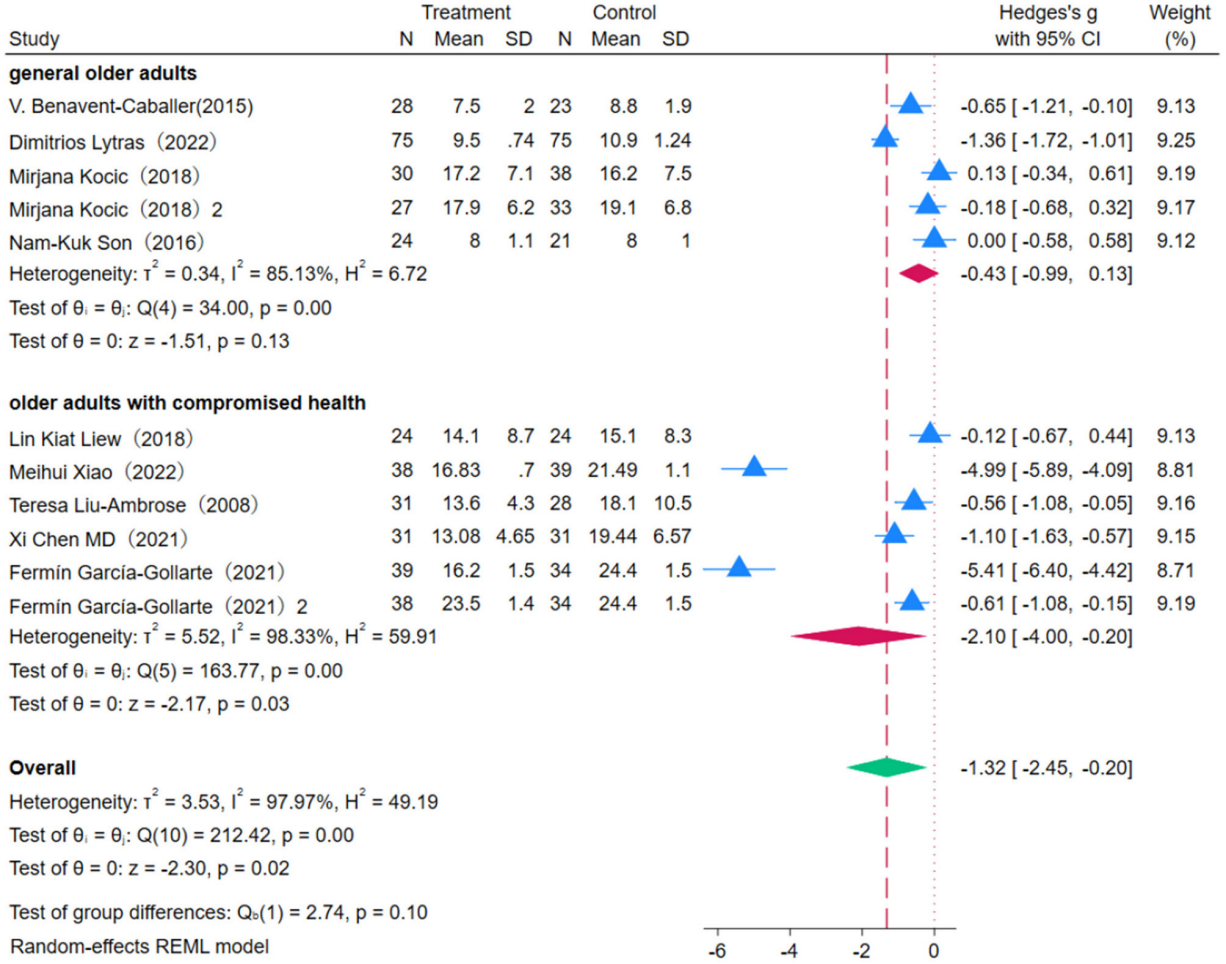
The impact of the OEP on mobility in older adults.

Nine studies were included to evaluate the effect of OEP on the mobility of older adults, using the Timed Up and Go (TUG) test as the assessment metric. Given the low heterogeneity observed (*P* = 0.00, *I*^2^ = 97.97%), a fixed-effects model was used in the meta-analysis (Figure 13). According to the findings, OEP significantly improved mobility in older individuals [WMD = −1.32, 95% CI (−2.45, −0.20)]. Compared to general older adults [WMD = −0.43, 95% CI (−0.99, 0.13)], the improvement in mobility was more pronounced in older adults with compromised health [WMD = −2.10, 95% CI (−4.00, −0.20)]. However, the wide confidence intervals that included zero for the general older adults group indicate that the effect was not statistically significant. Thus, OEP may be particularly beneficial for older adults with compromised health, as it can significantly improve their mobility. For general older adults, further adjustments or enhancements to interventions may be necessary to achieve significant effects. Despite the high heterogeneity in the meta-analysis, sensitivity analysis showed that the exclusion of most studies did not significantly alter the effect size estimates or confidence intervals, indicating robust results in this meta-analysis. Egger's regression intercept test indicated significant publication bias (*p* = 0.001). To further assess the stability of the results, the Trim-and-Fill method was applied, identifying and imputing two potentially missing studies. The adjusted effect size remained statistically significant, suggesting that while some degree of publication bias may be present, the core findings remain robust.

Subgroup analysis revealed that OEP did not significantly impact the mobility of older adults across varied intervention durations and session lengths. It is recommended that more standardized, high-quality studies be conducted to clarify the actual impact of different intervention durations.

### 3.8 The impact of the OEP on improving upper limb strength in older adults

**Figure 14:**
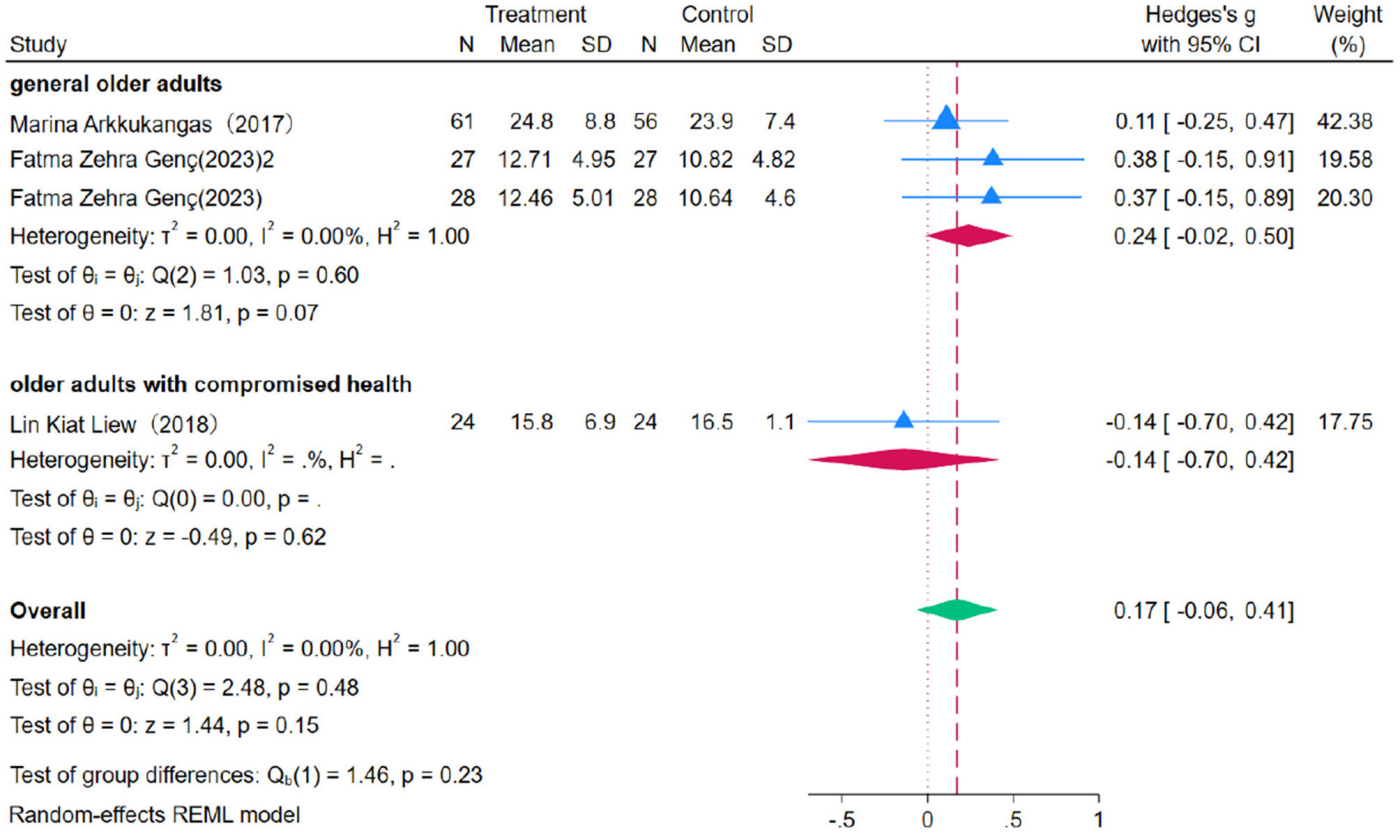
The impact of the OEP on right hand grip strength in older adults.

**Figure 15:**
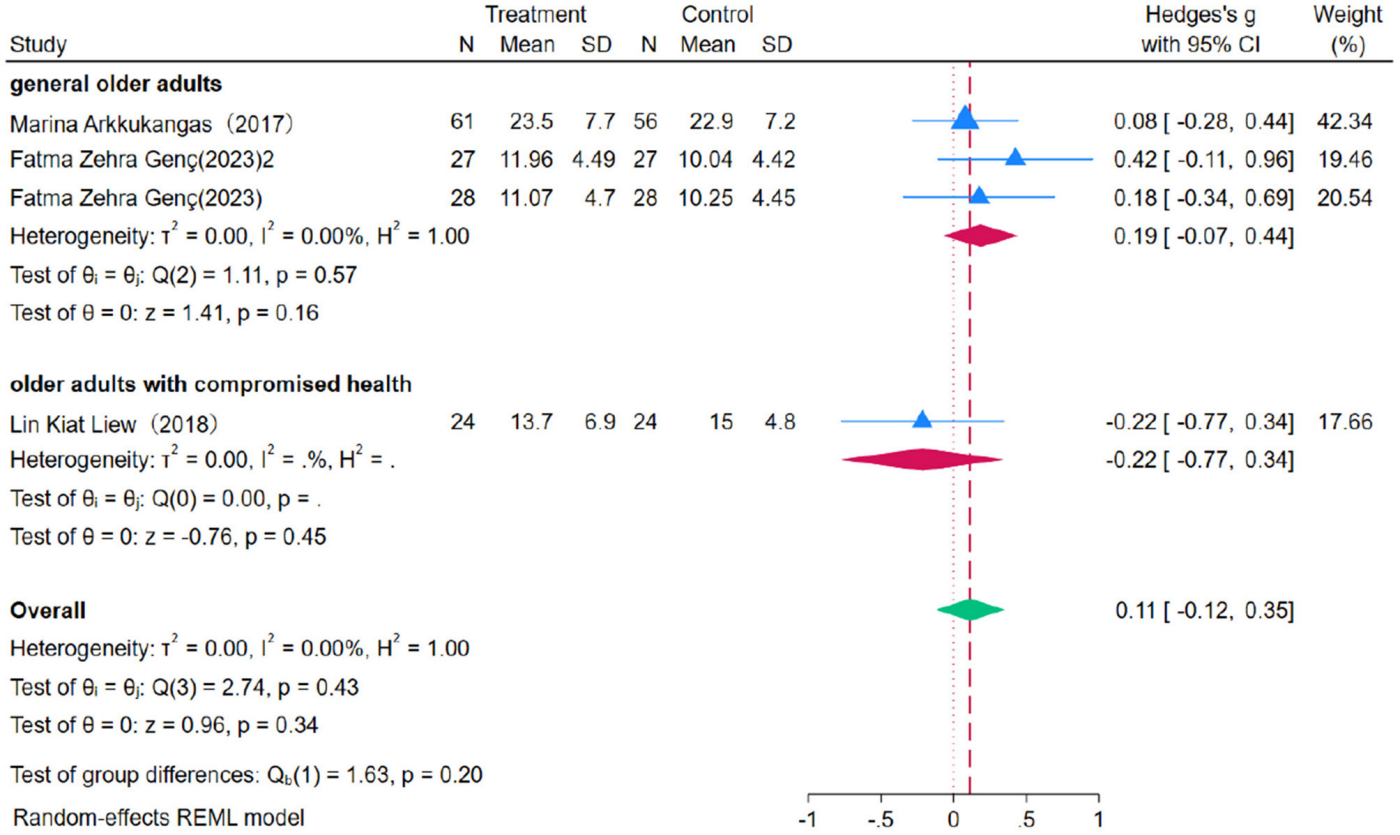
The impact of the OEP on left hand grip strength in older adults.

Nine studies were included to evaluate the impact of OEP on upper limb strength in older adults, using grip strength as the primary outcome measure. Due to minimal heterogeneity (*P* = 0.00, *I*^2^ = 0.00%), a fixed-effects model was applied for the meta-analysis (Figures 14, 15). The results indicated that the improvement in right-hand grip strength [WMD = 0.17, 95% CI (−0.06, 0.41)] and left-hand grip strength [WMD = 0.11, 95% CI (−0.12, 0.35)] was not statistically significant, as the confidence intervals included zero. Similarly, for general older adults, the improvement in right-hand grip strength [WMD = 0.24, 95% CI (−0.22, 0.50)] and left-hand grip strength [WMD = 0.19, 95% CI (−0.07, 0.44)] was not significant, nor was the improvement in right-hand [WMD = −0.14, 95% CI (−0.70, 0.42)] and left-hand grip strength [WMD = −0.22, 95% CI (−0.77, 0.34)] among older adults with compromised health. Therefore, the OEP does not appear to significantly improve upper limb strength in older adults. Egger's regression intercept test revealed no significant publication bias (right-hand grip strength *p* = 0.863, left-hand grip strength *p* = 0.981), indicating the reliability and representativeness of the results.

Due to the extremely low heterogeneity (I^2^= 0.00%) observed in the meta-analysis, a sensitivity analysis was not performed, as excluding individual studies is unlikely to impact the effect size estimates. The use of a fixed effects model further reinforces the robustness of the results. Additionally, Egger's regression intercept test indicated no significant publication bias (right-hand grip strength: *p* = 0.863; left-hand grip strength: *p* = 0.981), confirming the reliability and representativeness of the findings.

## 4 Discussion

This study conducted a meta-analysis to evaluate the impact of OEP on fall-related outcomes in both general older adults and older adults with compromised health. While OEP is designed to target fall susceptibility—a common risk factor among the older adults with compromised health—we acknowledge that variations in underlying health conditions may lead to subtle differences in intervention effectiveness. This study includes older adults with various conditions; however, the number of available studies was insufficient to conduct a meaningful subgroup analysis. Nonetheless, previous research has consistently demonstrated the effectiveness of OEP in reducing fall risk among individuals with cognitive impairment, musculoskeletal disorders, and frailty syndrome (19, 46, 47). Therefore, we analyzed these conditions collectively while acknowledging the potential for variations in intervention effects.

Although 17 included studies had a moderate to high risk of bias, their inclusion in the meta-analysis was essential to ensure a comprehensive synthesis of the available evidence. Excluding these studies would have significantly reduced the sample size, potentially introduced selection bias and limited the generalizability of the findings. To assess the impact of these methodological limitations, we conducted sensitivity analyses by systematically excluding these studies. The results remained consistent, confirming that their inclusion did not substantially alter the overall effect size estimates or confidence intervals. Additionally, Egger's regression test indicated significant reporting bias (*p* = 0.002), prompting the application of the Trim-and-Fill analysis to adjust for potential publication bias. The adjusted results remained statistically significant, further supporting the robustness of our conclusions. While certain studies exhibited biases in allocation concealment and blinding, their outcome assessments remained reliable, and these limitations were carefully considered in the interpretation of results. Therefore, despite inherent methodological concerns, the inclusion of these studies strengthens the overall analysis by maximizing available data, and sensitivity analyses affirm the stability and validity of our findings.

The results indicate that the OEP has a significant effect on improving balance, gait, and lower limb strength in older adults, although the extent of improvement varies among different populations. Consistent with previous studies, OEP has been shown to effectively enhance lower limb strength, balance, and gait in general older adults (13, 17, 48, 49). However, it does not significantly improve physical function, mobility, or upper limb strength in this population. Conversely, some studies have reported positive effects of OEP on physical function (43), mobility (23), and upper limb strength (50), highlighting the variability in outcomes across different study populations and intervention protocols. These discrepancies may be attributed to differences in participant characteristics, baseline functional levels, and variations in OEP implementation, including exercise intensity, frequency, and adherence rates.

As a fall prevention intervention, the OEP provides significant health benefits for general older adults, particularly in enhancing lower limb strength and balance. This effect may be attributed to specific exercises in the program, such as knee flexion, knee extension, and hip abduction, which are designed to strengthen leg muscles in older adults. Through cyclical contraction and relaxation, these exercises promote muscle and joint flexibility, balance, and motor control. Research has shown that the OEP is beneficial for older adults with osteoarthritis and gait balance disorders, significantly improving postural control (10).

Repeated exercises involving sitting, standing, and walking have been shown to improve lower limb strength in older adults (10). However, the results of this study indicate that its impact on physical function, mobility, and upper limb strength is not significant. Studies have shown that the OEP can improve physical function in older adults, including balance, lower limb strength, and mobility (23). This is inconsistent with the findings of the present study. This may be due to the OEP's primary focus on lower limb strength and balance training, which incorporates resistance and aerobic exercises through warm-up activities, strength training, balance exercises, and walking training (17). The program is heavily focused on lower limb strength and balance training, incorporating both resistance and aerobic exercises (28), which leads to higher intensity and frequency for lower limb and balance training. However, the upper limb strength training may not be sufficiently systematic or intense, thus failing to produce significant improvements.

This study demonstrates that OEP exhibits more significant positive effects on balance, gait, lower limb strength, and mobility in older adults with compromised health. This study demonstrates that the OEP has significant positive effects on balance, gait, lower limb strength, and mobility in older adults with compromised health.

High-quality evidence supports the effectiveness of the Otago Exercise Program in reducing fall risk among individuals with osteoarthritis (OA) (46). Additionally, OEP has been shown to benefit individuals with cognitive impairment, enhancing balance and functional mobility (51). These findings highlight OEP as a valuable intervention for older adults with varying health conditions, particularly those at higher risk of falls. The observed outcomes may be attributed to the lower baseline functional levels of older adults with compromised health, including reduced muscle strength, balance, and mobility. Additionally, these individuals often have stronger expectations for recovery, which may lead to higher engagement and adherence to rehabilitation training, thereby maximizing the benefits of OEP. Research has shown that OEP positively impacts balance, physical activity, and upper limb strength in older adults with reduced health status (19).

Our findings align with previous studies indicating that OEP can effectively mitigate the decline in lower limb muscle strength in older adults (22). The walking exercises included in the OEP—such as backward walking, zigzag walking, side walking, straight walking, and stair climbing—likely contribute to muscle strengthening and improved coordination through continuous isometric flexion of the hip flexors and ankle joints (22). The primary goal of the Otago Exercise Program is to reduce fall risk through targeted limb strength training and balance exercises (46). These diverse gait training exercises not only enhance lower limb muscle strength but also improve balance and gait control, thereby reducing fall risk and improving mobility in daily life. However, the results regarding upper limb strength contrast with those of other studies (43). In this study, OEP did not significantly improve upper limb strength in older adults with compromised health.

The primary goal of the OEP is fall prevention, with a focus on lower limb strength, balance, and gait training, rather than systematic upper limb strength exercises. This suggests that upper limb training is not a central component of the OEP. Additionally, older adults generally have lower baseline upper limb muscle strength, indicating that more targeted and intensive training may be required to achieve significant improvements. The OEP alone may not provide sufficient stimulus for upper limb muscle adaptation and strengthening, resulting in non-significant improvements in upper limb strength. Future research should explore incorporating dedicated upper limb strength training into the OEP to enhance overall physical function in older adults. Research indicates that lower upper limb strength is directly related to impaired activities of daily living (52) and slower walking speed (53). Thus, the lack of significant improvements in physical function and the limited effects on mobility observed in this study can be attributed to this.

Additionally, the results of the subgroup analysis indicate that differences exist in study design, sample selection, data collection, and analytical methods across studies (54). The duration of the intervention has varying effects on different outcomes. Specifically, a 4-month intervention demonstrated the most stable effects on balance improvement, while the effectiveness of 3-month and 6-month interventions showed high heterogeneity. Our findings align with previous research suggesting that a training program lasting at least 12 weeks, conducted three or more times per week, with each session lasting 30 to 45 minutes, can significantly reduce fall risk and enhance postural stability (45). However, no significant differences were found between different intervention durations (*p* = 0.30), indicating that the effectiveness of OEP may not be solely dependent on the intervention length. This is consistent with the findings of the present study.

As research suggests, sessions lasting more than 30 min are most effective in enhancing balance among older adults (55). Based on these findings, a 45-min session duration sustained for at least six months is recommended. Conversely, shorter intervention periods (2–3 months) and 30-min sessions appear to be more effective for improving lower limb strength. This may be because balance and gait primarily rely on neuromuscular coordination (56), which typically requires long-term practice and repetition for significant improvements. In contrast, lower limb strength training primarily targets large muscle groups, which require adequate recovery time post-exercise. Therefore, shorter intervention durations and 30-minute training sessions may be more suitable for lower limb strengthening, as they help prevent overtraining and muscle fatigue.

From a clinical perspective, these findings reinforce the importance of OEP as an effective intervention for maintaining lower limb strength and mobility in aging populations. The progressive nature of OEP exercises, such as knee flexion, knee extension, and hip abduction, may contribute to improved walking ability, reduced fall risk, and enhanced functional independence in older adults. Given that longer intervention durations (≥6 months) were associated with greater improvements in lower limb strength and gait, clinical practice should consider extending the program duration to maximize benefits. Additionally, incorporating upper limb resistance training into OEP may enhance its overall effectiveness, particularly for older adults with functional limitations. Future research should explore the integration of combined training approaches and assess their impact on overall musculoskeletal health in aging populations.

Although this study provides valuable insights into the effects of OEP intervention, it has certain limitations. The relatively small sample size may reduce statistical power, potentially limiting the generalizability and reliability of the findings. Moreover, the effectiveness of OEP may be influenced by various factors, including participants' health status, age, and baseline functional levels. Some included studies did not specify the MMSE criteria used to define cognitive impairment, which may have introduced variability in participant classification and contributed to heterogeneity across studies. Given that different older adult populations may respond differently to OEP, there is a need for more personalized training approaches. In this study, older adults with varying health conditions (e.g., cognitive impairment, musculoskeletal disorders, and frailty syndrome) were categorized into a single compromised health group. Previous research (19, 46, 47) has consistently demonstrated the effectiveness of OEP in reducing fall risk across these populations, supporting its broad applicability. While these individuals share a common risk factor—fall susceptibility, which OEP is specifically designed to address—differences in underlying health conditions may contribute to subtle variations in intervention effectiveness.

However, due to the limited number of available studies, subgroup analyses were not feasible. Future research should consider conducting stratified analyses to further explore whether OEP's effectiveness varies across specific health conditions. Furthermore, future studies should aim to optimize OEP protocols by tailoring them to individuals' health profiles, baseline fitness levels, and personal preferences to maximize intervention effectiveness.

## 5 Conclusion

OEP has been associated with improvements in balance, gait, and lower limb strength in older adults, particularly among those with compromised health, where the effects appeared more pronounced. These findings suggest that OEP may be particularly beneficial for enhancing lower limb strength and improving balance and gait. However, its impact on upper limb strength remains limited. Future research with larger, well-controlled trials is essential to validate these findings and refine intervention protocols to maximize their effectiveness in diverse aging populations.

## Data Availability

The original contributions presented in the study are included in the article/Supplementary material, further inquiries can be directed to the corresponding author/s.
