# Supplementary material for: The Otago Exercise Program's effect on fall prevention: a systematic review and meta-analysis
Source: Front Public Health. 2025 Jun 3;13:1522952. doi: 10.3389/fpubh.2025.1522952 (PMC12172654; doi:10.3389/fpubh.2025.1522952)
Supplement: Supplementary file 1 [file Supplementary_file_1.pdf]

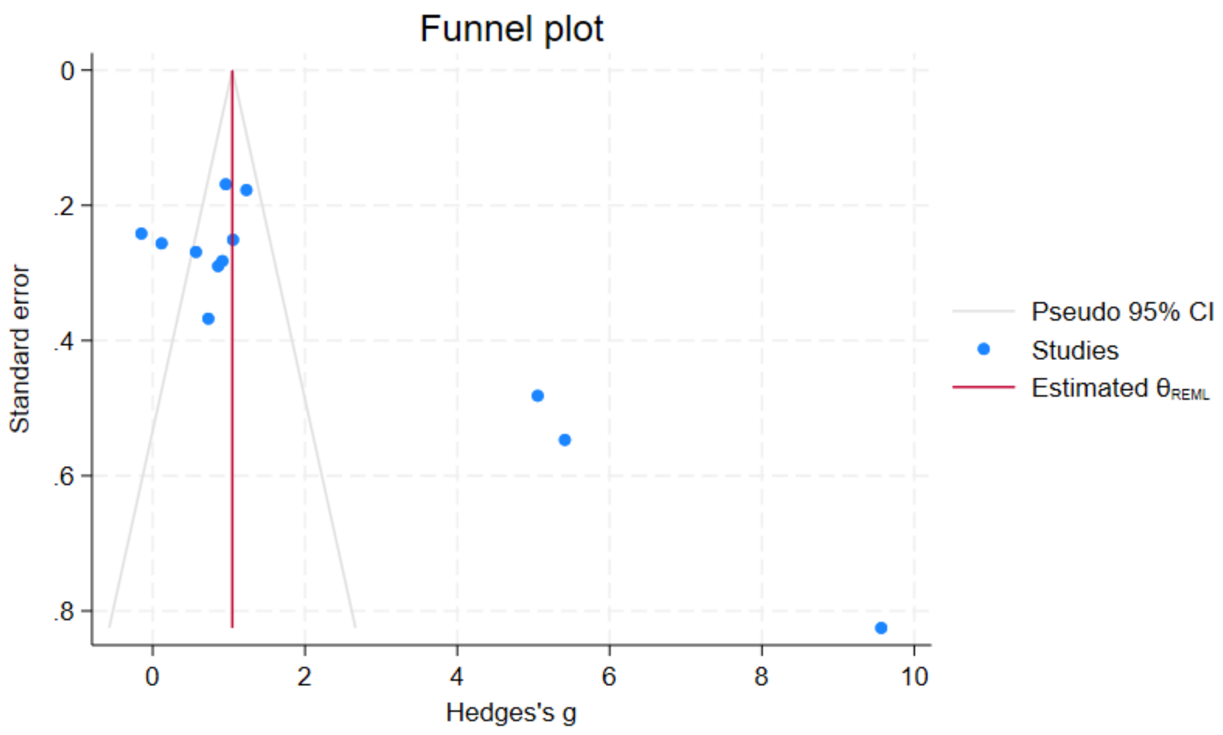

Figure 1 balance

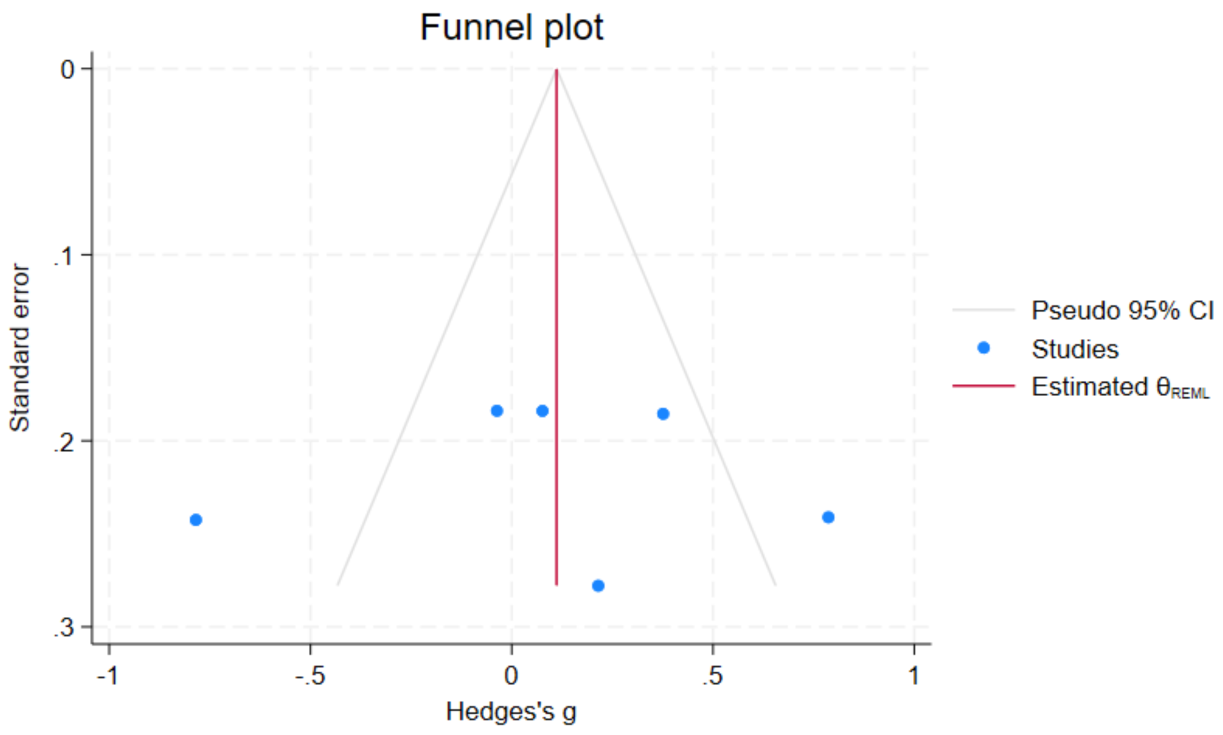

Figure 2 physical function

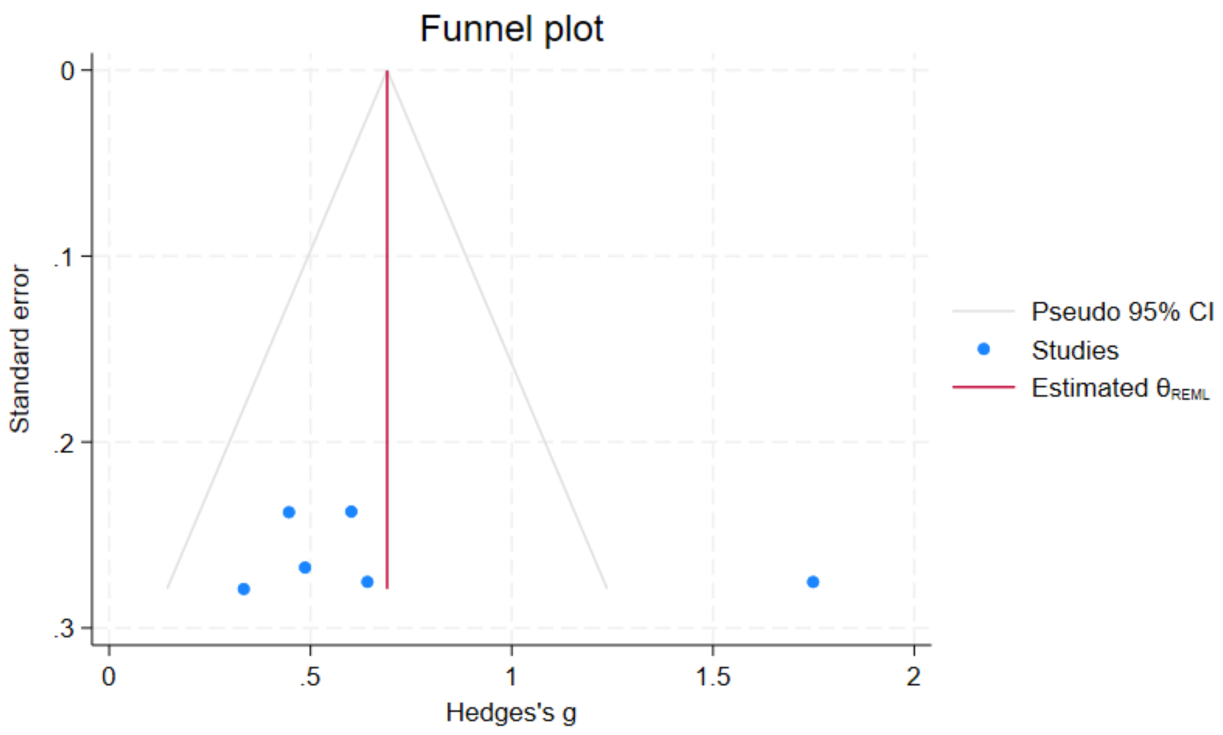

Figure 3 gait

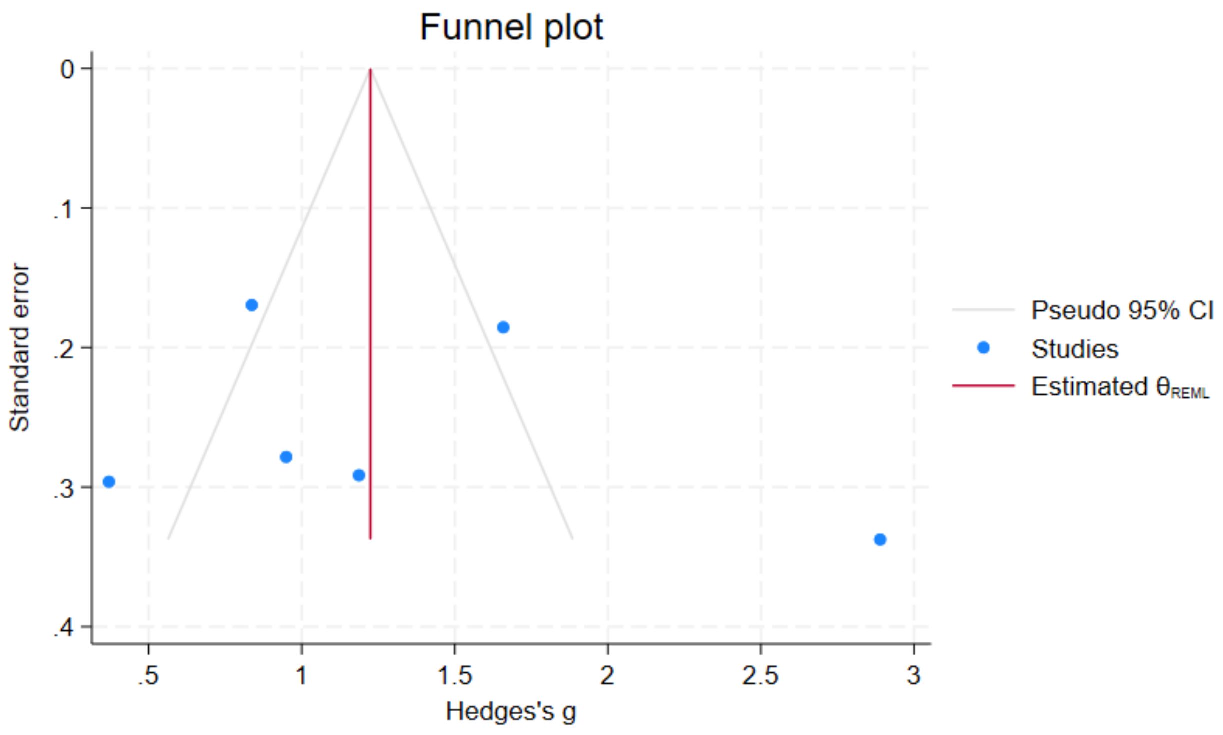

Figure 4 lower limb strength

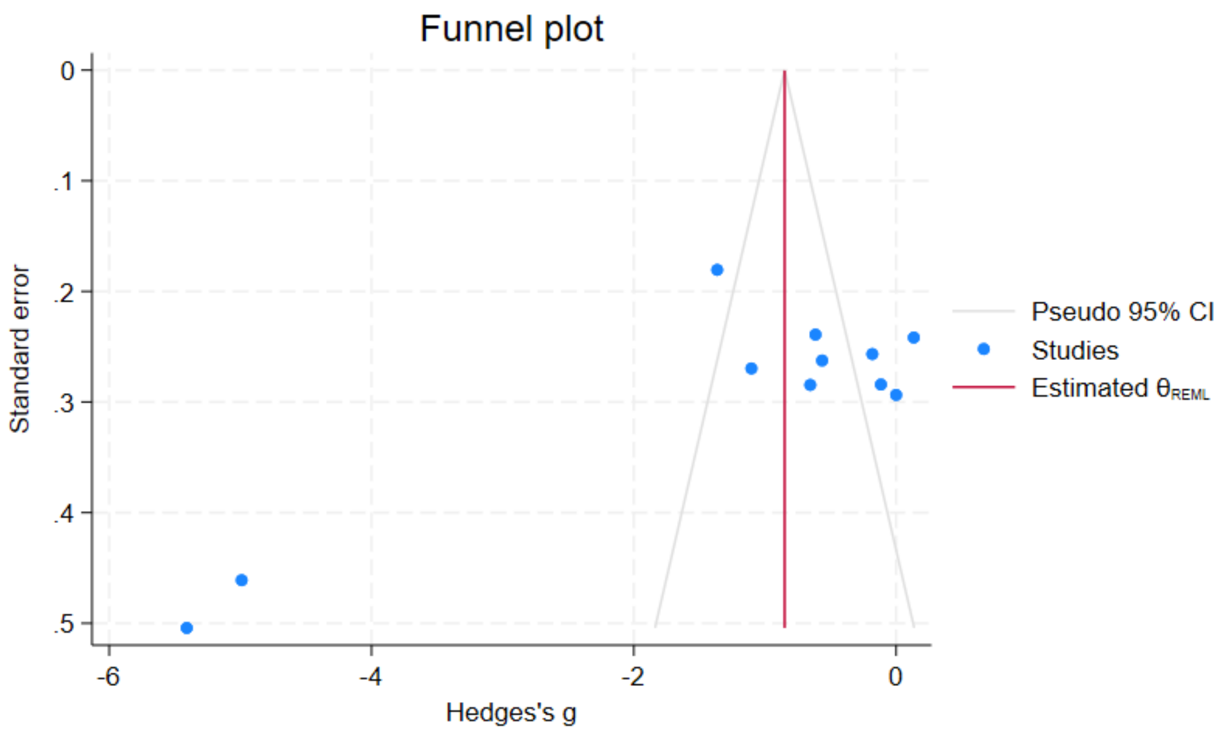

Figure 5 Mobility

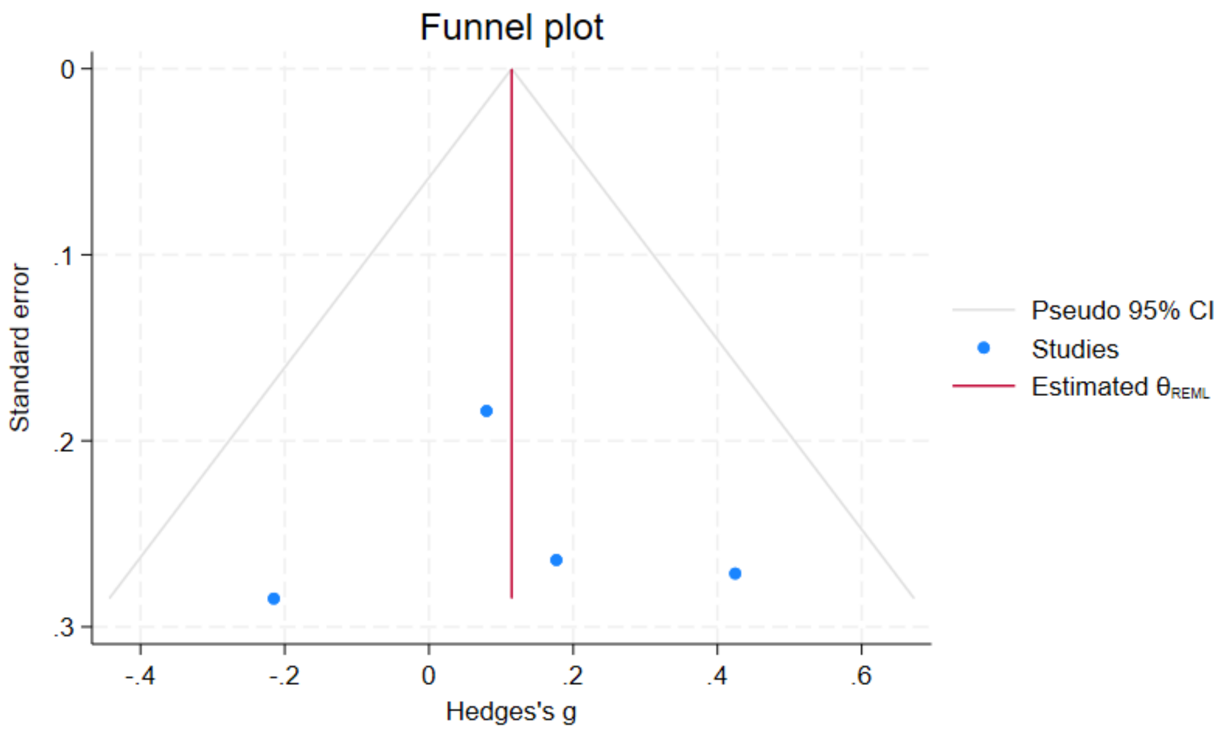

Figure 6 Right Hand Grip Strength

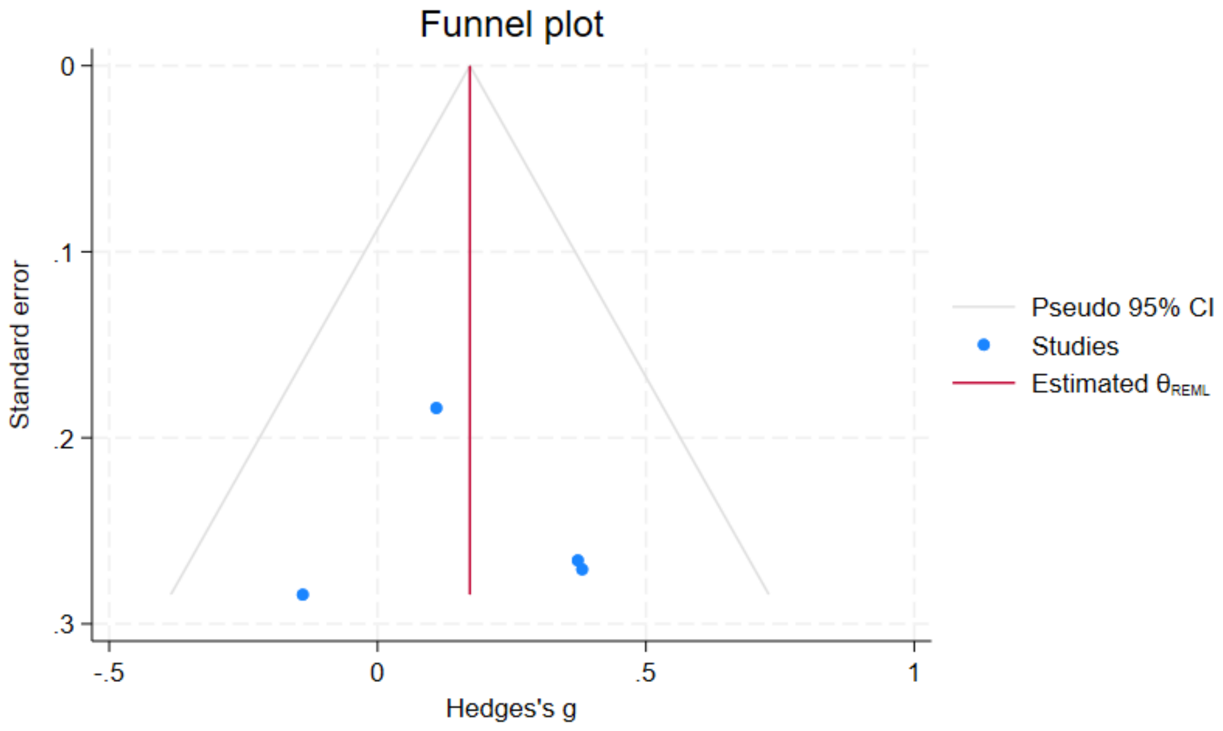

Figure 7 Left Hand Grip Strength
